# Identification of a genetic variant at 2q12.1 associated with blood pressure in East-Asians by genome-wide scan including gene-environment interactions

**DOI:** 10.1186/1471-2350-15-65

**Published:** 2014-06-05

**Authors:** Yun Kyoung Kim, Youngdoe Kim, Mi Yeong Hwang, Kazuro Shimokawa, Sungho Won, Norihiro Kato, Yasuharu Tabara, Mitsuhiro Yokota, Bok-Ghee Han, Jong Ho Lee, Bong-Jo Kim

**Affiliations:** 1 Division of Structural and Functional Genomics, Center for Genome Science, National Institute of Health, Centers for Disease Control and Prevention, 363-700 Chungcheongbuk-do, Republic of Korea; 2 Department of Food and Nutrition, College of Human Ecology, Yonsei University, Seoul, Republic of Korea; 3 Department of Gene Diagnostics and Therapeutics Research Institute, National Center for Global Health and Medicine, Tokyo, Japan; 4 Department of Applied Statistics, Chung-Ang University, Seoul, Republic of Korea; 5 Center for Genomic Medicine, Kyoto University Graduate School of Medicine, Kyoto, Japan; 6 Department of Genome Science, Aichi-Gakuin University, School of Dentistry, Nagoya, Japan

**Keywords:** Blood pressure, Genome-wide scan, Gene-environment interaction, Meta-analysis, Obesity

## Abstract

**Background:**

Genome-wide association studies have identified many genetic loci associated with blood pressure (BP). Genetic effects on BP can be altered by environmental exposures via multiple biological pathways. Especially, obesity is one of important environmental risk factors that can have considerable effect on BP and it may interact with genetic factors. Given that, we aimed to test whether genetic factors and obesity may jointly influence BP.

**Methods:**

We performed meta-analyses of genome-wide association data for systolic blood pressure (SBP) and diastolic blood pressure (DBP) that included analyses of interaction between single nucleotide polymorphisms (SNPs) and the obesity-related anthropometric measures, body mass index (BMI), height, weight, and waist/hip ratio (WHR) in East-Asians (n = 12,030).

**Results:**

We identified that rs13390641 on 2q12.1 demonstrated significant association with SBP when the interaction between SNPs and BMI was considered (*P* < 5 × 10 ^-8^). The gene located nearest to rs13390641, TMEM182, encodes transmembrane protein 182. In stratified analyses, the effect of rs13390641 on BP was much stronger in obese individuals (BMI ≥ 30) than non-obese individuals and the effect of BMI on BP was strongest in individuals with the homozygous A allele of rs13390641.

**Conclusions:**

Our analyses that included interactions between SNPs and environmental factors identified a genetic variant associated with BP that was overlooked in standard analyses in which only genetic factors were included. This result also revealed a potential mechanism that integrates genetic factors and obesity related traits in the development of high BP.

## Background

High blood pressure (BP) is one of the most common diseases worldwide and is an important risk factor for cardiovascular and renal disease [1,2]. Homeostasis of blood volume, blood vessel resistance and blood thickness are important for regulation of arterial pressure and these are maintained by complex interactions of several physiological pathways, including hormonal responses, nervous system signaling and intracellular feedback [3,4]. Variation in BP also reflects genetic factors with heritability ranging from 30 to 60% [5,6]. Large numbers of genetic variants associated with BP and hypertension have been identified in genome-wide association studies (GWASs) [7-11], but these common variants (minor allele frequency > 5%) with relatively small individual effect sizes for BP cannot fully explain the phenotypic variance [7]. To account for much of the heritability of complex traits, greater emphasis is being placed in recent years on gene-environment interaction analyses [12]. Interactions involving multiple genes and environmental factors underlying the biological network can potentially elucidate at least part of the missing heritability [13].

High BP develops from a complex interplay of genetic susceptibility factors and environmental factors [14]. A variety of environmental factors have been shown to influence BP, including obesity, physical inactivity, alcohol intake, tobacco use, and diet [1,3]. Obesity, in particular, is a main cause of high BP because it induces sympathoactivation that may raise BP [15].

To better understand the interactions between genes and environmental risk factors for BP, some candidate gene searches and few GWASs have included gene-environment interaction terms [16-18]. By using this method in GWAS, incorporation of genetic variations and environmental risk factors may yield additional novel loci that would not appear from analyses based on genetic effect only.

To examine this hypothesis in our study, we performed meta-analyses of GWASs for BP that included interactions between single nucleotide polymorphisms (SNPs) and the obesity-related anthropometric measures of body mass index (BMI), height, weight, and waist-hip ratio (WHR) using 12,030 East-Asians.

## Methods

### Study subjects

For our discovery stage and replication stage 1 study subjects were enlisted from those enrolled in the Korean Genome Epidemiology Study (KoGES) population-based cohort. We selected 7,486 subjects from the Korea Association REsource (KARE) project of KoGES [11] for the discovery stage and 3,703 subjects from the Health Examinee (HEXA) cohort [19] for replication stage 1. KARE project included the initial subjects composed of 10,038 individuals, aged 40 to 69, who were recruited from the Ansung and the Ansan regional cohorts that located in Gyeonggi province, near Seoul, the capital of Korea. Among them, only study subjects who had not been treated for hypertension, thyroid gland disease, osteoporosis, or asthma and who had not taken steroids, oral contraceptives, female sex hormone, or diuretics were included in the baseline for the discovery stage. Subjects of HEXA cohort were randomly selected from 1,200,000 participants of KoGES, aged 40 to 69, for use as a shared control in genome-wide disease association studies. More detailed explanations of both cohorts were previously described [11,19]. All subjects provided written informed consent and this study was approved by ethical committee of the institute (Korea Centers for Disease Control and Prevention Institutional Review Board).

To validate selected SNPs identified in the discovery and replication stage 1, we performed analyses in replication stage 2 using data collected from a total of 841 subjects enrolled in two independent studies of Japanese populations, the Amagasaki and Ehime studies, described previously [8].

### Phenotype determination

In discovery stage and replication stage 1, BP measurements were conducted using a standard mercury sphygmomanometer after the subjects had been in a sitting position for at least 5 min. In case of replication stage 2, BP was measured by automatic cuff-oscillometric device. The average of two measurements (left and right arm) was taken as the BP. The average of SBPs and DBPs of discovery stage, replication stage 1 and 2 are summarized in Table 1.

**Table 1:** Descriptive statistics of study samples

**Traits**		**Discovery**	**Replication 1**	**Replication 2**		* ***P***
		**KARE (n = 7,486)**	**HEXA (n = 3,703)**	**Ehime (n = 356)**	**Amagasaki (n = 485)**	
Age		51.4 ± 8.77	53.2 ± 8.33	61.3 ± 11.2	61.6 ± 6.97	< 0.0001
Gender	male (%)	3744 (50.0%)	1651 (44.6%)	126 (35.4%)	260 (53.6%)	-
	female (%)	3742 (50.0%)	2052 (55.4%)	230 (64.6%)	225 (46.3%)	-
Blood pressure	SBP (mmHg)	119.6 ± 17.4	121.7 ± 14.4	129.2 ± 19.7	128.4 ± 20.8	< 0.0001
	DBP (mmHg)	79.3 ± 11.1	77.1 ± 9.89	74.8 ± 11.0	77.2 ± 11.8	< 0.0001
Anthropometric measures	Height (cm)	160.5 ± 8.62	161.5 ± 8.10	158.0 ± 8.52	160.1 ± 7.97	< 0.0001
	Weight (kg)	63.0 ± 10.1	62.6 ± 9.97	56.8 ± 10.6	59.5 ± 10.2	0.076
	BMI (kg/m^2^)	24.4 ± 3.07	24.0 ± 2.90	22.6 ± 2.96	23.1 ± 3.13	< 0.0001
	WHR	0.88 ± 0.07	0.86 ± 0.07	-	-	< 0.0001

n, sample size; SBP, systolic blood pressure; DBP, diastolic blood pressure; BMI, body mass index; WHR, waist-hip ratio.

KARE, Korean Association Resource Project; HEXA, Health Examinee cohort.

Data are shown as mean ± standard deviation. *P values were analyzed by t-test between the two groups, KARE and HEXA.

The obesity-related anthropometric measures BMI, height, weight and WHR, were used in this study as environmental risk factors for elevated BP. BMI was defined as weight in kg divided by the square of height in m. WHR was defined as the ratio of waist (cm) to hip (cm) circumferences. In replication stage 2, only 3 of 4 anthropometric measures were available, BMI, height, weight except WHR.

### Genotyping and quality control

All of the 10,004 KARE study samples were genotyped using the Affymetrix Genome-Wide Human SNP array 5.0. After quality control, 8,842 subjects and 352,228 SNPs remained for analyses. For in silico replication, 4,302 individuals from the HEXA cohort were genotyped using the Affymetrix Genome-Wide Human SNP array 6.0. After quality control, 3,703 samples and 646,062 SNPs remained. Genotype calling methods and quality control criteria for samples and SNPs of both cohorts have been previously described [11,19].

SNP imputation was conducted using the IMPUTE program [20] based on International HapMap (phase 2, release 22, NCBI build 36 and dbSNP build 126; http://hapmap.ncbi.nlm.nih.gov/) data from JPT and CHB populations. We used 1,573,409 SNPs for the KARE study and 1,984,393 SNPs for the HEXA cohort after excluding imputed SNPs of unsatisfactory quality for genetic analyses [19].

The results for replication stage 2 were generated from 356 individuals from the Ehime study using the Illumina Human Omni 2.5-8 BeadChip and 485 individuals from the Amagasaki study using the Illumina HumanHap 550 k Quad BeadChip.

### Statistical analyses

Standardized residuals of SBP and DBP adjusted for age and sex by linear regression were used as the phenotypes for analyses in each study. To investigate the effect of interaction between SNPs and the anthropometric measures for BP, we conducted linear regression analyses with interaction terms based on the equation: Y = β_0_ + β_1_ × SNP + β_2_ × anthropometric measures (BMI, height, weight, WHR) + β_3_ × (SNP × anthropometric measures). Y is the residual of SBP or DBP, β_0_ is a constant, β_1_ and β_2_ are the main effect of a particular SNP and a particular anthropometric measure, respectively, and β_3_ is the effect of the interaction term being tested. All of analyses in each stage were conducted using the R program (version 2.15.2; http://www.r-project.org/).

SNPs were selected for replication stage 1 if the SNP’s main effect P value was less than 1 × 10^-4^ in the discovery stage. SNPs that were found to be statistically significant (*P*_SNP_ < 0.05) in replication stage 1 were selected for replication stage 2. To combine association results for selected SNPs in multiple stages (discovery, replication 1 and replication 2), we performed inverse variance weighted meta-analysis for each SNP’s main and interactional effects from each stage using the rmeta package of the R program in which fixed effects were assumed.

After meta-analyses, SNPs with the accepted genome-wide significance level (*P* < 5 × 10^-8^), which reflected testing of one million SNPs [21], were considered statistically significant. The more conservative genome-wide significance threshold is *P* < 3.18 × 10^-8^ based on Bonferroni correction, but no SNP in this study exceeded this threshold. It should be noted that we have selected SNPs which showed the moderate signal (*P*_SNP_ < 1.0 × 10^-4^) in the discovery stage, expected to be achieved by abundant genetic variants. This stage would require less correction for multiple testing than the final stage targeting at genome-wide significance [22]. From our 3-stage study design, we have discovered a genetic variation that reached genome-wide significance in the final stage.

We also performed stratified analyses to identify the combined effect of BMI and SNP on BP. We tested the association between BMI and BP in each genotype of SNP (ex: GG, GA, AA) and the association between SNP and BP in each BMI sub-group (BMI < 18.5, 18.5 ≤ BMI < 25, 25 ≤ BMI < 30, BMI ≥ 30).

### Prediction of TFBS using ENCODE database

To elucidate the biological meaning of variant, we examined the cluster scores of transcription factor binding sites (TFBS) nearby SNP based on data from all five ENCODE (The Encyclopedia of DNA Elements Consortium) TFBS ChIP-seq production groups via UCSC Genome Browser (http://genome.ucsc.edu/). The UCSC Genome Browser has released a track containing 690 datasets of transcription factor ChIP-seq peaks.

## Results

### Descriptive information of study samples

Table 1 provides the descriptive characteristics of the 12,030 study subjects enlisted for the discovery stage of our analysis from the Korea Association REsource (KARE) project of the Korean Genome Epidemiological Study (KoGES), for replication stage 1 from the Health Examinee cohort (HEXA) of KoGES, and for replication stage 2 from Japanese populations enrolled in the Ehime and Amagasaki studies [8,11,19]. The HEXA group had a higher proportion of women (56.6%) than the KARE group (50.0%), and the average SBP was higher in the HEXA subjects (121.7 ± 14.4) than in the KARE subjects (119.6 ± 17.4). In contrast, the average DBP was higher in the KARE subjects (79.3 ± 11.1) than in the HEXA subjects (77.1 ± 9.89). All but one (weight) of variables in two groups showed statistically significant differences (*P* < 0.0001). The Japanese populations examined in replication stage 2 tended to be older and to have higher SBP than the HEXA and KARE groups. The correlations between BP and anthropometric measures are summarized in Additional file 1: Table S1. There were moderate to low correlations between BP and anthropometric measures across the KARE and HEXA subjects (Pearson’s r = |0.1 ~ 0.3|). But, most of the differences between two independent correlation coefficients were statistically significant (Additional file 1: Table S1).

### A genome-wide scan including interaction terms

**Figure 1:**
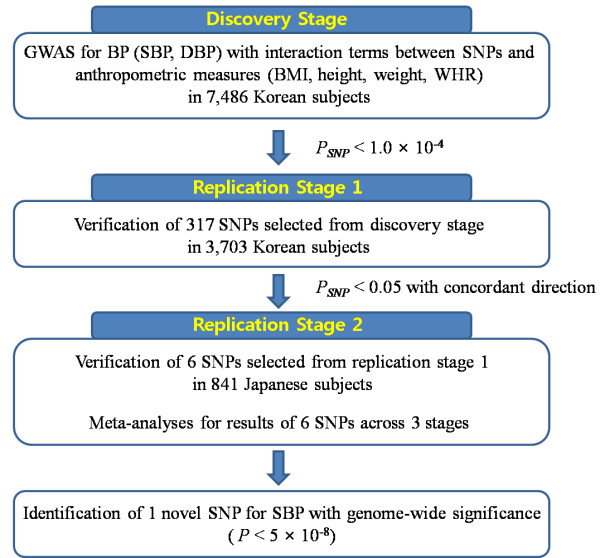
**Overall study scheme.** We carried out a genome-wide scan for BP-associated (SBP and DBP) SNPs that included interaction terms between SNPs and the anthropometric measures BMI, height, weight, and WHR. After the three-stage analysis (discovery, replication 1, replication 2), we identified a SNP that was strongly associated with SBP by a linear regression model that incorporated interaction between SNPs and BMI.

We performed genome-wide association analyses on SBP and DBP in the 7,486 KARE subjects with consideration of interaction between approximately 1.6 million SNPs and four obesity-related anthropometric measures using four linear regression models with the following interaction terms: SNP × BMI, height, weight, or WHR (See Figure 1 for the overall study scheme). Genomic inflation factors (λ_gc_) of each SNP’s main and interactional effects were calculated for each model. All of the λ_gc_ values were ≤ 1.057 (Additional file 1: Table S2). The quantile-quantile plots for SBP and DBP in each model with interaction terms are presented in Additional file 1: Figure S1.

In discovery stage, we identified several BP associated genetic loci with moderate significance (*P*_SNP_ < 1.0 × 10^-4^) including some that had not been detected in previous BP-associated GWASs which considered only the SNP’s main effects when analyzed (Additional file 1: Table S3). To verify these loci in subsequent stage, we selected the 317 SNPs exceeding *P*_SNP_ < 1.0 × 10^-4^ that showed the most significant signals at each locus considering Linkage Disequilibrium block (LD, r^2^ > 0.2) for replication stage 1 with 3,703 HEXA subjects, 146 SNPs associated with SBP only, 150 SNPs associated with DBP only and 21 associated with both SBP and DBP (Additional file 1: Table S3).

In replication stage 1, we found six SNPs that showed statistical significance (*P*_SNP_ < 0.05) in the SNP’s main effect with the concordant direction in discovery stage’s. These six SNPs were also tested in Japanese populations (n = 841) of replication stage 2 (Additional file 1: Table S4).

In inverse variance meta-analyses combining the results from the three stages for the six SNPs (Additional file 1: Table S4), there was one SNP that reached genome-wide significance (*P* < 5 × 10^-8^). SNP rs13390641, located in the intergenic region on 2q12.1 near the transmembrane protein 182 gene (TMEM182), was found to be associated with SBP when the interaction between SNP and BMI was considered in the analyses (combined *P*_SNP_ = 3.83 × 10^-8^ and *P*_INT_ = 5.28 × 10^-8^; Table 2). For rs13390641, there was no evidence for heterogeneity across the three stages (heterogeneity *P* = 0.47; Table 2).

**Table 2:** SNP with a significant effect on BP when considering interaction between SNP and BMI of the subject

**Trait**	**Interaction**	**SNP; cytoband; nearby genes; minor allele/major allele**	*Type of effect	**Discovery (n = 7,486)**			**Replication 1 (n = 3,703)**			**Replication 2 (n = 841)**			**Combined (n = 12,030)**		
				**MAF**	**Beta (se)**	***P***	**MAF**	**Beta (se)**	***P***	**MAF**	**Beta (se)**	***P***	**Beta (se)**	***P***	**P**_**het **_**(Q)**
SBP	BMI	^#^rs13390641; 2q12.1; TMEM182; A/G	SNP main	0.11	-13.9(3.35)	3.51 × 10^–5^	0.10	-11.6 (4.35)	7.73 × 10^–3^	0.10	-32.1 (12.5)	1.01 × 10^–2^	-14.4 (2.62)	3.83 × 10^–8^	0.47 (2.52)
			Interaction		0.56 (0.14)	3.80 × 10^–5^		0.47 (0.18)	9.39 × 10^–3^		1.35 (0.54)	1.21 × 10^–2^	0.59 (0.11)	5.28 × 10^–8^	0.48 (2.49)

^#^rs13390641 is an imputed SNP in each stage and the effect allele is A; *Type of effect in a linear regression model with SNP × BMI interaction term; SBP, systolic blood pressure; BMI, body mass index; MAF, minor allele frequency; Beta, standardized regression coefficient; se, standard error.

A test of heterogeneity (P_het_) was conducted; Q, Cochrane’s Q value based on chi-squared statistics.

Age and sex were covariates in this analysis.

### Comparison of the effect of rs13390641 and BMI on BP in categorized groups

**Figure 2:**
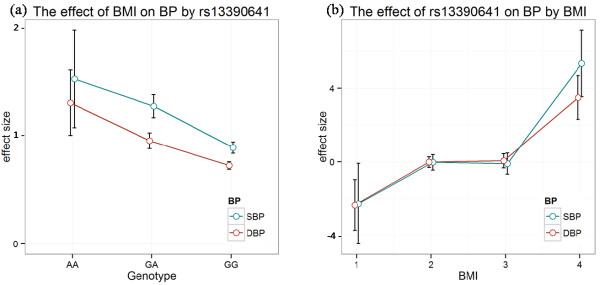
**Effect size of BMI and rs13390641 on BP. (a)** Effect of BMI on SBP and DBP in the three rs13390641 genotypes. **(b)** Effect of rs13390641 on SBP and DBP in the four BMI groups. Group 1: BMI < 18.5, Group 2: 18.5 ≤ BMI < 25, Group 3: 25 ≤ BMI < 30, and Group 4: BMI ≥ 30. Data are the mean ± standard deviation.

We compared the effect estimates for BMI on BP among three genotype classes of rs13390641 by linear regression analyses of the KARE and HEXA subjects (n = 11,189). The strongest effect of BMI was for SBP in subjects carrying the homozygous A allele of rs13390641 (Beta = 1.58, *P* = 9.68 × 10^-4^). In the case of DBP, the effect of BMI was also highest in subjects carrying the homozygous A allele (Beta = 1.35, *P* = 3.26 × 10^-5^; Table 3 and Figure 2).

**Table 3:** Effect of BMI on BP by rs13390641 genotype classes

**Trait**	**Genotype**	**n**	**Beta (se)**	***P***
SBP	GG	8980	0.92 (0.05)	4.62 × 10^-66^
	GA	2051	1.32 (0.11)	5.76 × 10^-32^
	AA	135	1.58 (0.47)	9.68 × 10^-4^
DBP	GG	8980	0.75 (0.04)	7.81 × 10^-96^
	GA	2051	0.99 (0.07)	2.02 × 10^-39^
	AA	135	1.35 (0.31)	3.26 × 10^-5^

SBP, systolic blood pressure; DBP, diastolic blood pressure; n, sample size of each subgroup; Beta, standardized regression coefficient; se, standard error.

The association analyses between BP and BMI in each group were performed using linear regression adjusted by age and sex.

We also calculated effect estimates for rs13390641 on BP in four BMI sub-groups. In the highest BMI sub-group (BMI ≥ 30), rs13390641 showed a strong increasing effect on SBP (Beta = 5.35, *P* = 3.22 × 10^-3^). For DBP, the effect of rs13390641 also increased significantly in the highest BMI sub-group (Beta = 3.50, *P* = 3.24 × 10^-3^; Table 4 and Figure 2). It is enough to say that the subjects in the highest BMI sub-group with carrying A allele may have a higher risk of increasing BP significantly.

**Table 4:** Effect of rs13390641 on BP by BMI sub-groups

**Trait**	**BMI sub-group**	**n**	**Beta (se)**	***P***
SBP	BMI < 18.5	212	-2.26 (2.18)	0.2993
	18.5 ≤ BMI < 25	6700	-0.02 (0.43)	0.9540
	25 ≤ BMI < 30	3850	-0.10 (0.58)	0.8690
	30 ≤ BMI	427	5.35 (1.81)	0.0032
DBP	BMI < 18.5	212	-2.34 (1.37)	0.0884
	18.5 ≤ BMI < 25	6700	-0.01 (0.29)	0.9600
	25 ≤ BMI < 30	3850	0.06 (0.39)	0.8720
	30 ≤ BMI	427	3.50 (1.18)	0.0032

SBP, systolic blood pressure; DBP, diastolic blood pressure; BMI, body mass index; n, sample size of each subgroup; Beta, standardized regression coefficient; se, standard error; The association analyses between BP and rs13390641 in each group were performed using linear regression adjusted by age and sex.

## Discussion

Few studies have examined the potential interaction between SNPs and environmental risk factors that modulate complex traits on a genome-wide scale. The major strength of our study was that it was able to detect a previously overlooked BP-associated genetic factor, rs13390641, with genome-wide significance (*P* < 5 × 10^-8^) because our analyses considered interaction between SNPs and obesity related anthropometric measures.

The estimated effect of BMI on BP was larger in individuals carrying two copies of the A allele of rs13390641 than in those carrying two copies of the G allele (Table 3) and the effect of rs13390641 on BP was larger in obese individuals (BMI ≥ 30) than in the other subjects (Table 4). These stratified analyses strongly suggest that the joint effect of rs13390641 and obesity (BMI) on risk of developing BP identified that the effect in the obese individuals carrying risk (A) alleles (GA or AA genotype) was much higher than that in obese individuals carrying non-risk (G) alleles.

The rs13390641 SNP is located intergenic region, 602 kb downstream of the gene that encodes transmembrane protein 182, which is composed of four putative membrane-spanning regions [23]. There were low correlation between variants in TMEM182 and rs13390641 (Pearson’s correlation coefficient, r ≈ 0.10) (Additional file 1: Figure S2). We also examined LD block between variants in TMEM182 and rs13390641 using Haploview software based on HapMap release 22, CHB + JPT panel. These variants located in a same locus, 2q12.1, but were not in a LD (Additional file 1: Figure S3). However, interestingly, there were several transcription factor binding sites located between TMEM182 and rs13390641. Of them, binding sites for RAD21, CTCF and STAT1 showed the highest cluster scores that were 767, 500 and 769, respectively (Visualization in the UCSC Genome Browser, http://genome.ucsc.edu/). Especially, STAT1 plays a part in the process of blood circulation (referring to the Gene Ontology). It may affect to regulate the expression of TMEM182 and suggests a possible modulation of BP level.

No phenotypes with a direct functional connection to TMEM182 have been reported, but its expression in adipocytes and its potential role in glaucoma have been explored [23,24]. Elevated levels of the proinflammatory cytokine TNF-α are associated with obesity [25,26]. TNF-α also regulates the expression of TMEM182 in white adipose tissue [23]. Taken together, these results suggest that expression of TMEM182 may be integral to the adipocyte phenotype mediated by the TNF-α pathway, and that TMEM182 may act on BP.

Up to date, some studies have discovered genetic variants associated BP via GWAS using KARE subjects [27-30]. These studies have identified 8 genes, PARK2, OPA1, ATP2B1, CSK, ARSG, CSMD1, CYP17A1 and PLEKHA7 that located SNPs associated BP or hypertension. We have tested genetic effects of 22 SNPs on 8 genes for SBP considered interaction between SNPs and BMI. However, none of them represented the statistical significance in this interaction model (Additional file 1: Table S5). It is due to method of analyses that previous studies conducted standard analyses which considered only genetic effects, on the other hand, our analyses considered not only genetic effects but also interactional effects. According to the different method, it is natural that the result came out differently. So, these previously known SNPs associated BP based on KARE subjects were not to be considered as candidates for BP in our analyses.

Although we uncovered only a single locus with significant genome-wide association with BP, possibly because of genetic diversity and differences in environmental effects between populations, this is the first study to examine genome-wide associations of BP with interacting genetic and environmental risk factors in East-Asian populations. Incorporating gene-environment interactions may be critical for discovering genetic determinants of complex traits such as BP, as these interactions may better explain the variance of complex traits than direct effects of individual genes.

## Conclusions

In conclusion, this genome-wide screen for BP-associated genes, which considered interaction between SNPs and obesity-related anthropometric measures, identified a genetic variant in East-Asian populations near TMEM182 that may influence BP. This study suggests a useful strategy for discovering new genetic contributors of complex traits, which may work together with environmental factors to cause human diseases.

## Competing interests

The authors declare that they have no competing interests.

## Authors’ contributions

YKK provided the design of the project and performed data analysis, writing and editing manuscript. YK and MYH participated in data analysis, KS carried out data analysis in replication stage 2. SW reviewed data, and NK, YT and MY provided the data of Japanese population. B-GH, JHL and B-JK participated in general discussion, reviewed data and editing manuscript. All authors read and approved the final manuscript.

## Pre-publication history

The pre-publication history for this paper can be accessed here:

http://www.biomedcentral.com/1471-2350/15/65/prepub

## Supplementary Material

Additional file 1: Figure S1 The quantile - quantile plots for SBP and DBP in each model with interaction terms in discovery stage. **Figure S2.** Pearson’s correlation coefficient (r) between rs13390641 and 206 SNPs located from *TMEM182* to rs13390641 (Chr 2:102744010 – 103402865). **Figure S3.** The linkage disequilibrium (LD) block between rs13390641 and SNPs on *TMEM182*. **Table S1.** Correlation of BP and anthropometric measures. **Table S2.** Genomic inflation factors of analyses in each model with interaction terms in the discovery stage. **Table S3.** Results of genome-wide association analyses in discovery stage. **Table S4.** Results of combined meta-analyses for selected 6 SNPs. **Table S5.** Results of association analyses for SBP considered interaction between BMI and known SNPs that were revealed by earlier experiences within the KARE project. Click here for file

## References

[B1] Whitworth JA 2003 World Health Organization (WHO)/International Society of Hypertension (ISH) statement on management of hypertension J Hypertens 2003 15 11 1983 1992 14597836 10.1097/00004872-200311000-00002

[B2] Wadei HM Textor SC The role of the kidney in regulating arterial blood pressure Nat Rev Nephrol 2012 15 10 602 609 10.1038/nrneph.2012.191 22926246

[B3] Padmanabhan S Newton-Cheh C Dominiczak AF Genetic basis of blood pressure and hypertension Trends Genet 2012 15 8 397 408 10.1016/j.tig.2012.04.001 22622230

[B4] Staessen JA Wang J Bianchi G Birkenhager WH Essential hypertension Lancet 2003 15 9369 1629 1641 10.1016/S0140-6736(03)13302-8 12747893

[B5] Hottenga JJ Boomsma DI Kupper N Posthuma D Snieder H Willemsen G de Geus EJ Heritability and stability of resting blood pressure Twin Res Hum Genet 2005 15 5 499 508 10.1375/twin.8.5.499 16212839

[B6] Kupper N Willemsen G Riese H Posthuma D Boomsma DI de Geus EJ Heritability of daytime ambulatory blood pressure in an extended twin design Hypertension 2005 15 1 80 85 10.1161/01.HYP.0000149952.84391.54 15557390

[B7] Ehret GB Munroe PB Rice KM Bochud M Johnson AD Chasman DI Smith AV Tobin MD Verwoert GC Hwang SJ Pihur V Vollenweider P O'Reilly PF Amin N Bragg-Gresham JL Teumer A Glazer NL Launer L Zhao JH Aulchenko Y Heath S Sõber S Parsa A Luan J Arora P Dehghan A Zhang F Lucas G Hicks AA Jackson AU Genetic variants in novel pathways influence blood pressure and cardiovascular disease risk Nature 2011 15 7367 103 109 10.1038/nature10405 21909115 PMC3340926

[B8] Kato N Takeuchi F Tabara Y Kelly TN Go MJ Sim X Tay WT Chen CH Zhang Y Yamamoto K Katsuya T Yokota M Kim YJ Ong RT Nabika T Gu D Chang LC Kokubo Y Huang W Ohnaka K Yamori Y Nakashima E Jaquish CE Lee JY Seielstad M Isono M Hixson JE Chen YT Miki T Zhou X Meta-analysis of genome-wide association studies identifies common variants associated with blood pressure variation in east Asians Nat Genet 2011 15 6 531 538 10.1038/ng.834 21572416 PMC3158568

[B9] Adeyemo A Gerry N Chen G Herbert A Doumatey A Huang H Zhou J Lashley K Chen Y Christman M Rotimi C A genome-wide association study of hypertension and blood pressure in African Americans PLoS Genet 2009 15 7 e1000564 10.1371/journal.pgen.1000564 19609347 PMC2702100

[B10] Newton-Cheh C Johnson T Gateva V Tobin MD Bochud M Coin L Najjar SS Zhao JH Heath SC Eyheramendy S Papadakis K Voight BF Scott LJ Zhang F Farrall M Tanaka T Wallace C Chambers JC Khaw KT Nilsson P van der Harst P Polidoro S Grobbee DE Onland-Moret NC Bots ML Wain LV Elliott KS Teumer A Luan J Lucas G Genome-wide association study identifies eight loci associated with blood pressure Nat Genet 2009 15 6 666 676 10.1038/ng.361 19430483 PMC2891673

[B11] Cho YS Go MJ Kim YJ Heo JY Oh JH Ban HJ Yoon D Lee MH Kim DJ Park M Cha SH Kim JW Han BG Min H Ahn Y Park MS Han HR Jang HY Cho EY Lee JE Cho NH Shin C Park T Park JW Lee JK Cardon L Clarke G McCarthy MI Lee JY Lee JK A large-scale genome-wide association study of Asian populations uncovers genetic factors influencing eight quantitative traits Nat Genet 2009 15 5 527 534 10.1038/ng.357 19396169

[B12] Manolio TA Collins FS Cox NJ Goldstein DB Hindorff LA Hunter DJ McCarthy MI Ramos EM Cardon LR Chakravarti A Cho JH Guttmacher AE Kong A Kruglyak L Mardis E Rotimi CN Slatkin M Valle D Whittemore AS Boehnke M Clark AG Eichler EE Gibson G Haines JL Mackay TF McCarroll SA Visscher PM Finding the missing heritability of complex diseases Nature 2009 15 7265 747 753 10.1038/nature08494 19812666 PMC2831613

[B13] Eichler EE Flint J Gibson G Kong A Leal SM Moore JH Nadeau JH Missing heritability and strategies for finding the underlying causes of complex disease Nat Rev Genet 2010 15 6 446 450 10.1038/nrg2809 20479774 PMC2942068

[B14] Pausova Z Tremblay J Hamet P Gene-environment interactions in hypertension Curr Hypertens Rep 1999 15 1 42 50 10.1007/s11906-999-0072-z 10981041

[B15] Pausova Z From big fat cells to high blood pressure: a pathway to obesity-associated hypertension Curr Opin Nephrol Hypertens 2006 15 2 173 178 10.1097/01.mnh.0000214775.42103.a5 16481885

[B16] Taylor JY Sun YV Hunt SC Kardia SL Gene-environment interaction for hypertension among African American women across generations Biol Res Nurs 2010 15 2 149 155 10.1177/1099800410371225 20591971 PMC3005771

[B17] Tsuchihashi-Makaya M Serizawa M Yanai K Katsuya T Takeuchi F Fujioka A Yamori Y Ogihara T Kato N Gene-environmental interaction regarding alcohol-metabolizing enzymes in the Japanese general population Hypertens Res 2009 15 3 207 213 10.1038/hr.2009.3 19262484

[B18] Sabatti C Service SK Hartikainen AL Pouta A Ripatti S Brodsky J Jones CG Zaitlen NA Varilo T Kaakinen M Sovio U Ruokonen A Laitinen J Jakkula E Coin L Hoggart C Collins A Turunen H Gabriel S Elliot P McCarthy MI Daly MJ Järvelin MR Freimer NB Peltonen L Genome-wide association analysis of metabolic traits in a birth cohort from a founder population Nat Genet 2009 15 1 35 46 10.1038/ng.271 19060910 PMC2687077

[B19] Kim YJ Go MJ Hu C Hong CB Kim YK Lee JY Hwang JY Oh JH Kim DJ Kim NH Kim S Hong EJ Kim JH Min H Kim Y Zhang R Jia W Okada Y Takahashi A Kubo M Tanaka T Kamatani N Matsuda K Park T Oh B Kimm K Kang D Shin C Cho NH MAGIC consortium Large-scale genome-wide association studies in East Asians identify new genetic loci influencing metabolic traits Nat Genet 2011 15 10 990 995 10.1038/ng.939 21909109

[B20] Marchini J Howie B Myers S McVean G Donnelly P A new multipoint method for genome-wide association studies by imputation of genotypes Nat Genet 2007 15 7 906 913 10.1038/ng2088 17572673

[B21] Moskvina V Schmidt KM On multiple-testing correction in genome-wide association studies Genet Epidemiol 2008 15 6 567 573 10.1002/gepi.20331 18425821

[B22] Pe’er I Yelensky R Altshuler D Daly MJ Estimation of the multiple testing burden for genomewide association studies of nearly all common variants Genet Epidemiol 2008 15 4 381 385 10.1002/gepi.20303 18348202

[B23] Wu Y Smas CM Expression and regulation of transcript for the novel transmembrane protein Tmem182 in the adipocyte and muscle lineage BMC Res Notes 2008 15 85 10.1186/1756-0500-1-85 18803820 PMC2564950

[B24] Shi D Funayama T Mashima Y Takano Y Shimizu A Yamamoto K Mengkegale M Miyazawa A Yasuda N Fukuchi T Abe H Ideta H Nishida K Nakazawa T Richards JE Fuse N Association of HK2 and NCK2 with normal tension glaucoma in the Japanese population PLoS One 2013 15 1 e54115 10.1371/journal.pone.0054115 23349798 PMC3551945

[B25] Ruan H Hacohen N Golub TR Van Parijs L Lodish HF Tumor necrosis factor-alpha suppresses adipocyte-specific genes and activates expression of preadipocyte genes in 3 T3-L1 adipocytes: nuclear factor-kappaB activation by TNF-alpha is obligatory Diabetes 2002 15 5 1319 1336 10.2337/diabetes.51.5.1319 11978627

[B26] Wellen KE Uysal KT Wiesbrock S Yang Q Chen H Hotamisligil GS Interaction of tumor necrosis factor-alpha- and thiazolidinedione-regulated pathways in obesity Endocrinology 2004 15 5 2214 2220 10.1210/en.2003-1580 14764635

[B27] Jin HS Hong KW Kim BY Kim J Yoo YH Oh B Jeong SY Replicated association between genetic variation in the PARK2 gene and blood pressure Clin Chim Acta 2011 15 17–18 1673 1677 21635879 10.1016/j.cca.2011.05.026

[B28] Jin HS Sober S Hong KW Org E Kim BY Laan M Oh B Jeong SY Age-dependent association of the polymorphisms in the mitochondria-shaping gene, OPA1, with blood pressure and hypertension in Korean population Am J Hypertens 2011 15 10 1127 1135 10.1038/ajh.2011.131 21796221

[B29] Hong KW Go MJ Jin HS Lim JE Lee JY Han BG Hwang SY Lee SH Park HK Cho YS Oh B Genetic variations in ATP2B1, CSK, ARSG and CSMD1 loci are related to blood pressure and/or hypertension in two Korean cohorts J Hum Hypertens 2010 15 6 367 372 10.1038/jhh.2009.86 19960030

[B30] Hong KW Jin HS Lim JE Kim S Go MJ Oh B Recapitulation of two genomewide association studies on blood pressure and essential hypertension in the Korean population J Hum Genet 2010 15 6 336 341 10.1038/jhg.2010.31 20414254

