# Supplementary material for: Identification of a genetic variant at 2q12.1 associated with blood pressure in East-Asians by genome-wide scan including gene-environment interactions
Source: BMC Med Genet. 2014 Jun 5;15:65. doi: 10.1186/1471-2350-15-65 (PMC4059884; doi:10.1186/1471-2350-15-65)
Supplement: Additional file 1: Figure S1 — The quantile - quantile plots for SBP and DBP in each model with interaction terms in discovery stage. Figure S2. Pearson’s correlation coefficient (r) between rs13390641 and 206 SNPs located from TMEM182 to rs13390641 (Chr 2:102744010 – 103402865). Figure S3. The linkage disequilibrium (LD) block between rs13390641 and SNPs on TMEM182. Table S1. Correlation of BP and anthropometric measures. Table S2. Genomic inflation factors of analyses in each model with interaction terms in the discovery stage. Table S3. Results of genome-wide association analyses in discovery stage. Table S4. Results of combined meta-analyses for selected 6 SNPs. Table S5. Results of association analyses for SBP considered interaction between BMI and known SNPs that were revealed by earlier experiences within the KARE project. [file 1471-2350-15-65-S1.doc]

**Additional File 1**

**Identification of a genetic variant at 2q12.1 associated with blood pressure in East-Asians by genome-wide scan including gene-environment interactions**

Yun Kyoung Kim^1,2^, Youngdoe Kim^1^, Mi Yeong Hwang^1^, Kazuro Shimokawa^3^, Sungho Won^4^, Norihiro Kato^3^, Yasuharu Tabara^5^, Mitsuhiro Yokota^6^, Bok-Ghee Han^1^, Jong Ho Lee^2^  and Bong-Jo Kim^1§^

*^1^Division of Structural and Functional Genomics, Center for Genome Science, National Institute of Health, Centers for Disease Control and Prevention, Chungcheongbuk-do, Korea, ^2^Department of Food and Nutrition, College of Human Ecology, Yonsei University, Seoul, Korea, ^3^Department of Gene Diagnostics and Therapeutics Research Institute, National Center for Global Health and Medicine, Tokyo, Japan, ^4^Department of Applied Statistics, Chung-Ang University, Seoul, Korea, ^5^Center for Genomic Medicine, Kyoto University Graduate School of Medicine, Kyoto, Japan, ^6^Department of Genome Science, Aichi-Gakuin University, School of Dentistry, Nagoya, Japan*

**Figure S1** The quantile - quantile plots for SBP and DBP in each model with interaction terms in discovery stage showed genome-wide distribution of the empirically observed of test statistic in comparison with the expected null distribution. (a) is plots for SBP and (b) is for DBP.


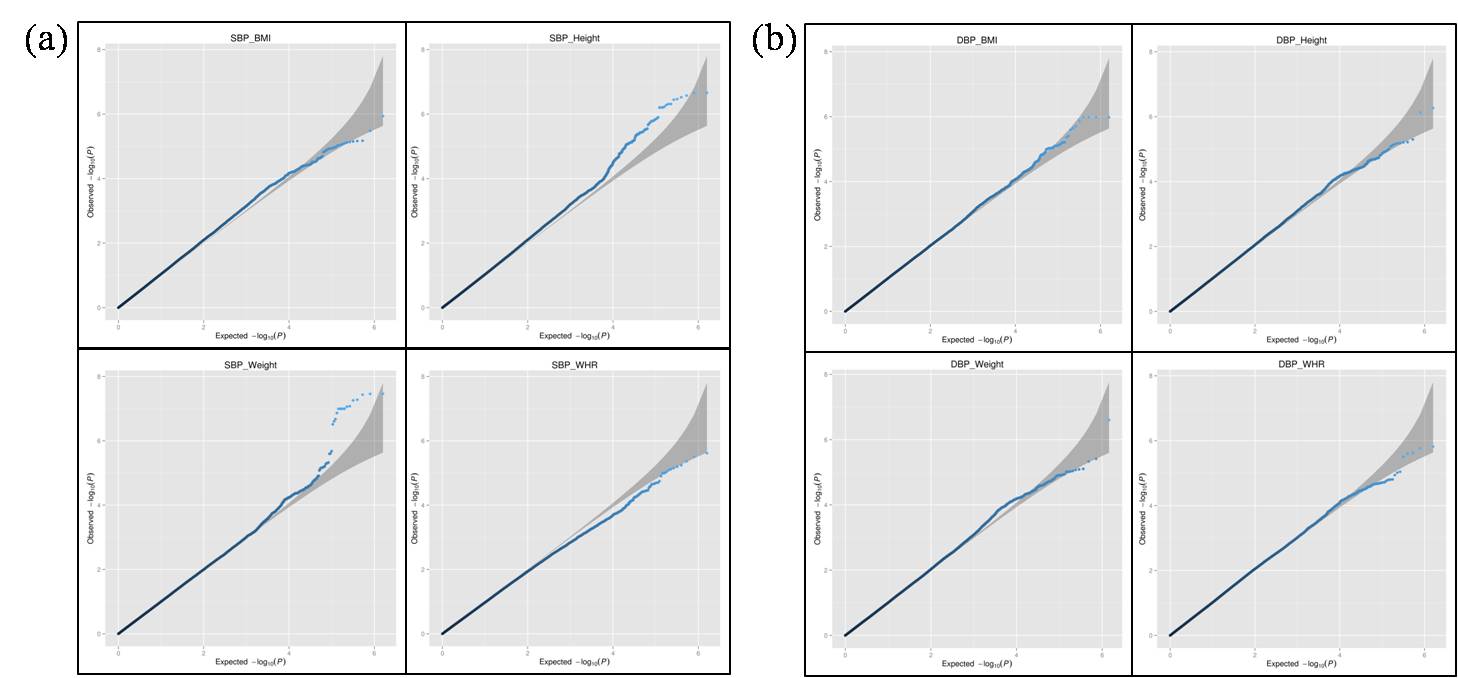


**Figure S2** Pearson’s correlation coefficient (r) between rs13390641 and 206 SNPs located from *TMEM182* to rs13390641 (Chr 2:102744010 – 103402865). Blue dots represent Pearson’s r absolute values. Because of the distance (602kb), there are low correlations between SNPs in *TMEM182* and rs13390641 (r ≈ 0.10).

**Figure S3** The linkage disequilibrium (LD) block between rs13390641 and SNPs on *TMEM182* is showed by HapMap data based on CHB + JPT. Red arrows represent the location of TMEM182 and rs13390641.


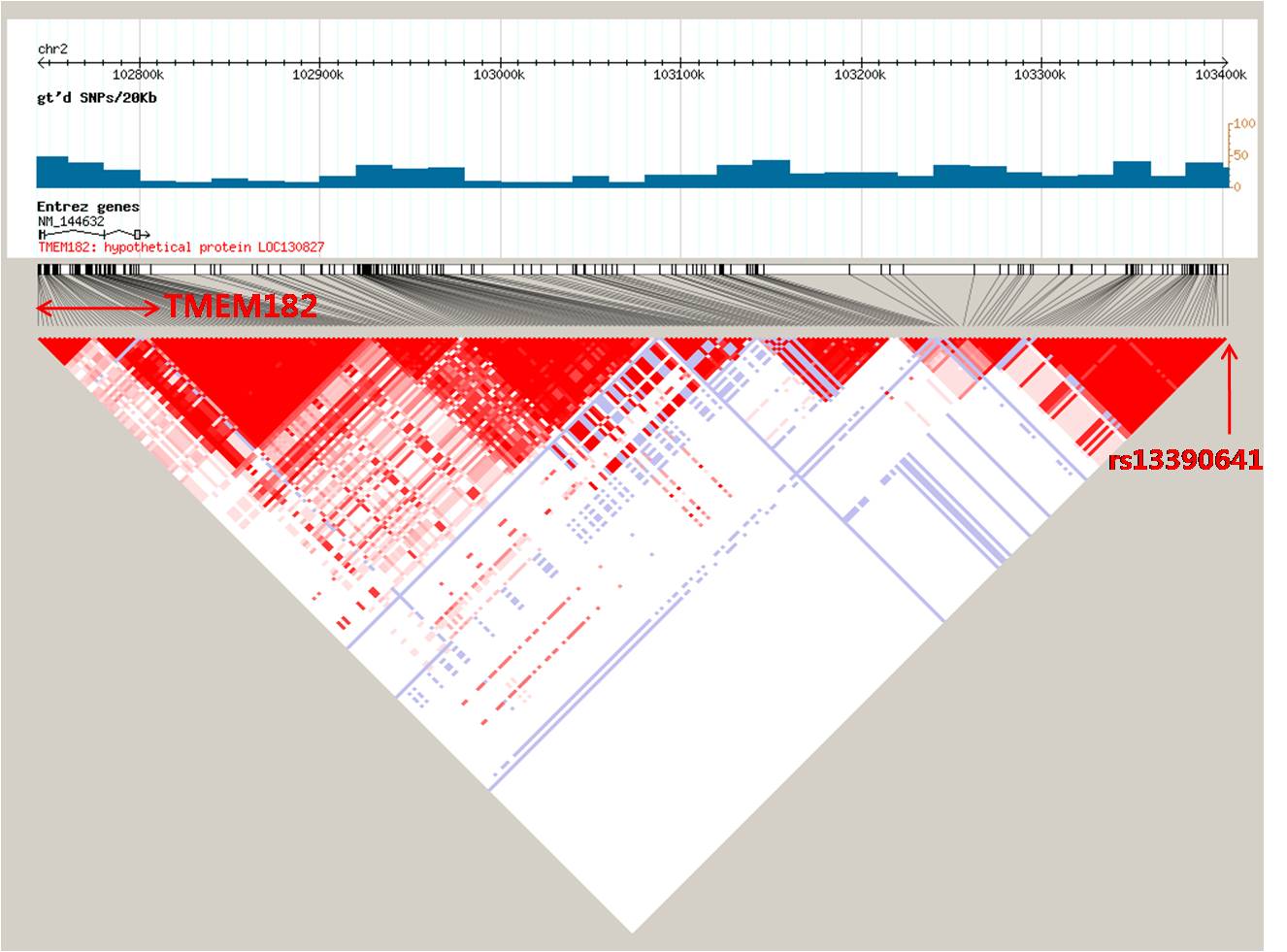


| **Table S1** Correlation of BP and anthropometric measures | | | | | | | | |
| --- | --- | --- | --- | --- | --- | --- | --- | --- |
| Trait | Discovery stage KARE (n=7,486) | |  | Replication stage 1 HEXA (n=3,703) | |  | **P* value | |
|  | SBP | DBP |  | SBP | DBP |  | SBP | DBP |
| BMI | 0.16 | 0.22 |  | 0.21 | 0.18 |  | 0.0099 | 0.0385 |
| Height | -0.05 | 0.08 |  | 0.12 | 0.16 |  | < 0.0001 | 0.0001 |
| Weight | 0.10 | 0.23 |  | 0.23 | 0.24 |  | < 0.0001 | 0.5961 |
| WHR | 0.32 | 0.31 |  | 0.23 | 0.19 |  | < 0.0001 | < 0.0001 |
| SBP, systolic blood pressure; DBP, diastolic blood pressure; BMI, body mass index; WHR, waist-hip ratio; | | | | | | | | |
| All of values in Table were represented as Pearson's correlation coefficient. | | | | | | | | |
| **P* values were calculated by the test, Fisher r-to-z transformation, of the difference between two independent correlation coefficients | | | | | | | | |

| **Table S2** Genomic inflation factors of analyses in each model with interaction terms in the discovery stage | | | |
| --- | --- | --- | --- |
| Trait | Interaction | SNP’s main | Interaction |
| SBP | BMI | 1.051 | 1.057 |
|  | Height | 1.032 | 1.021 |
|  | Weight | 1.003 | 0.978 |
|  | WHR | 0.967 | 0.982 |
| DBP | BMI | 1.011 | 1.021 |
|  | Height | 1.008 | 1.005 |
|  | Weight | 1.004 | 1.010 |
|  | WHR | 1.020 | 1.027 |
| SBP, systolic blood pressure; DBP, diastolic blood pressure; BMI, body mass index; WHR, waist-hip ratio; Genomic inflation factor is defined as the ratio of the median of the observed distribution of the test statistic to the expected median. | | | |
| SNP’s main is calculated using by statistics of SNP’s main effect and Interaction is calculated by statistics of interactional effect in a regression model. | | | |

| **Table S3** Results of genome-wide association analyses in discovery stage (KARE) (*P_SNP_* < 1 × 10 ^-4^) | | | | | | | |  |  |  |  |  |
| --- | --- | --- | --- | --- | --- | --- | --- | --- | --- | --- | --- | --- |
| **Trait** | **Interaction** | **SNP** | **CHR** | **Location(bp)** | **Cytoband** | **genes** | **Function** | **MA** | **KARE (n=7,486)** | | | |
|  |  |  |  |  |  |  |  |  | **^1^Beta(se)_SNP_** | ***^2^P_SNP_*** | **^3^Beta(se)_INT_** | ***^4^P_INT_*** |
| SBP | BMI | rs41528452 | 1 | 72253505 | 1p31.1 | NEGR1 | intronic | C | 19.6(4.82) | 5.01E-05 | -0.77(0.20) | 9.83E-05 |
|  |  | rs379230 | 1 | 82146683 | 1p31.1 | LPHN2 | intronic | T | -36.2(8.65) | 2.92E-05 | 1.50(0.35) | 1.98E-05 |
|  |  | rs498502 | 1 | 48497683 | 1p33 |  | intergenic | G | -8.74(2.21) | 8.02E-05 | 0.36(0.09) | 5.91E-05 |
|  |  | rs2268568 | 1 | 166552468 | 1q24.2 |  | intergenic | C | 9.01(2.28) | 7.81E-05 | -0.37(0.09) | 8.36E-05 |
|  |  | rs360089 | 1 | 224124954 | 1q42.12 | TMEM63A | intronic | T | 13.5(3.07) | 1.07E-05 | -0.51(0.13) | 4.02E-05 |
|  |  | rs4853891 | 2 | 2600693 | 2p25.3 |  | intergenic | A | 9.27(2.37) | 9.34E-05 | -0.36(0.10) | 1.79E-04 |
|  |  | rs13390641 | 2 | 103402865 | 2q12.1 |  | intergenic | G | -13.9(3.35) | 3.51E-05 | 0.56(0.14) | 3.80E-05 |
|  |  | rs291465 | 2 | 190891206 | 2q32.2 | HIBCH | intronic | C | 11.7(2.92) | 6.44E-05 | -0.45(0.12) | 1.30E-04 |
|  |  | rs9881523 | 3 | 55903019 | 3p14.3 | ERC2 | intronic | T | -10.1(2.50) | 5.19E-05 | 0.41(0.10) | 4.82E-05 |
|  |  | rs7630022 | 3 | 39090498 | 3p22.2 | WDR48 | intronic | G | 10.4(2.36) | 1.08E-05 | -0.43(0.10) | 6.69E-06 |
|  |  | rs9871349 | 3 | 3531273 | 3p26.2 |  | intergenic | A | -17.9(4.53) | 8.14E-05 | 0.74(0.18) | 5.31E-05 |
|  |  | rs1473485 | 3 | 113627419 | 3q13.2 |  | intergenic | T | 20.9(5.23) | 6.46E-05 | -0.85(0.21) | 6.33E-05 |
|  |  | rs7683961 | 4 | 42949646 | 4p13 |  | intergenic | T | -17.7(4.30) | 3.80E-05 | 0.75(0.17) | 1.42E-05 |
|  |  | rs13150373 | 4 | 16466871 | 4p15.32 | LDB2 | intronic | T | 28.9(7.42) | 9.76E-05 | -1.13(0.30) | 1.65E-04 |
|  |  | rs1506798 | 4 | 10795548 | 4p16.1 |  | intergenic | G | 9.35(2.39) | 9.34E-05 | -0.38(0.10) | 9.14E-05 |
|  |  | rs6816803 | 4 | 179756519 | 4q34.3 |  | intergenic | C | -12.5(2.95) | 2.23E-05 | 0.50(0.12) | 2.60E-05 |
|  |  | rs13172034 | 5 | 52060154 | 5q11.2 |  | intergenic | T | -10.3(2.35) | 1.24E-05 | 0.41(0.10) | 1.48E-05 |
|  |  | rs12186484 | 5 | 78189081 | 5q14.1 | ARSB | intronic | C | 9.63(2.35) | 4.36E-05 | -0.39(0.10) | 4.57E-05 |
|  |  | rs2925180 | 5 | 114704671 | 5q22.3 |  | intergenic | C | 9.52(2.40) | 7.36E-05 | -0.37(0.10) | 1.69E-04 |
|  |  | rs10055901 | 5 | 119424780 | 5q23.1 |  | intergenic | C | -9.14(2.22) | 3.74E-05 | 0.38(0.09) | 2.33E-05 |
|  |  | rs1106894 | 5 | 147033382 | 5q32 | JAKMIP2 | intronic | C | -9.35(2.08) | 7.07E-06 | 0.38(0.08) | 6.69E-06 |
|  |  | rs10056942 | 5 | 148396307 | 5q33.1 | SH3TC2 | intronic | C | 16.5(4.17) | 7.36E-05 | -0.67(0.17) | 8.36E-05 |
|  |  | rs6878732 | 5 | 156397834 | 5q33.3 | HAVCR1 | intronic | T | 11.8(2.89) | 4.21E-05 | -0.49(0.12) | 3.54E-05 |
|  |  | rs158894 | 5 | 167918852 | 5q35.1 | MIR103A1 | 3downstream | G | 17.0(4.22) | 5.72E-05 | -0.69(0.17) | 6.31E-05 |
|  |  | rs4711716 | 6 | 42375201 | 6p21.1 | TRERF1 | intronic | A | -12.5(2.99) | 3.06E-05 | 0.52(0.12) | 2.37E-05 |
|  |  | rs17586946 | 6 | 26047032 | 6p22.2 |  | intergenic | C | 17.5(4.13) | 2.31E-05 | -0.71(0.17) | 1.83E-05 |
|  |  | rs9496314 | 6 | 142637912 | 6q24.1 |  | intergenic | C | -36.1(8.34) | 1.50E-05 | 1.45(0.34) | 2.33E-05 |
|  |  | rs6570933 | 6 | 149455013 | 6q25.1 |  | intergenic | T | -9.34(2.35) | 7.03E-05 | 0.39(0.10) | 4.99E-05 |
|  |  | rs645106 | 7 | 4156439 | 7p22.2 | SDK1 | intronic | T | 10.5(2.39) | 1.17E-05 | -0.42(0.10) | 1.70E-05 |
|  |  | rs10227746 | 7 | 94325756 | 7q21.3 |  | intergenic | T | -27.2(6.56) | 3.39E-05 | 1.11(0.27) | 3.70E-05 |
|  |  | rs10242121 | 7 | 149816758 | 7q36.1 |  | intergenic | C | -8.57(2.13) | 5.61E-05 | 0.35(0.09) | 4.23E-05 |
|  |  | rs16910575 | 8 | 83601084 | 8q21.13 |  | intergenic | C | 8.51(2.04) | 3.09E-05 | -0.35(0.08) | 2.16E-05 |
|  |  | rs11993703 | 8 | 93847305 | 8q22.1 |  | intergenic | A | -18.1(4.54) | 6.56E-05 | 0.75(0.18) | 4.65E-05 |
|  |  | rs7006940 | 8 | 102559664 | 8q22.3 |  | intergenic | T | 10.3(2.30) | 7.31E-06 | -0.42(0.09) | 7.95E-06 |
|  |  | rs1904863 | 8 | 136137625 | 8q24.22 |  | intergenic | T | 8.76(2.18) | 5.79E-05 | -0.37(0.09) | 3.06E-05 |
|  |  | rs10968364 | 9 | 28162436 | 9p21.1 | LINGO2 | intronic | A | -12.3(3.00) | 4.21E-05 | 0.51(0.12) | 3.26E-05 |
|  |  | rs17737524 | 9 | 24682844 | 9p21.3 |  | intergenic | G | 28.4(7.04) | 5.49E-05 | -1.24(0.29) | 1.64E-05 |
|  |  | rs1339761 | 9 | 109739598 | 9q31.2 |  | intergenic | G | 38.2(8.48) | 6.84E-06 | -1.53(0.35) | 1.15E-05 |
|  |  | rs3004256 | 10 | 42785918 | 10q11.21 |  | intergenic | A | 11.4(2.89) | 7.54E-05 | -0.47(0.12) | 6.84E-05 |
|  |  | rs17098371 | 10 | 83497679 | 10q23.1 |  | intergenic | T | -42.5(10.3) | 3.97E-05 | 1.75(0.42) | 2.52E-05 |
|  |  | rs4244315 | 10 | 97232891 | 10q23.33 | SORBS1 | intronic | A | 27.9(6.62) | 2.62E-05 | -1.12(0.27) | 3.23E-05 |
|  |  | rs1530252 | 10 | 109331873 | 10q25.1 |  | intergenic | C | 9.12(2.18) | 2.82E-05 | -0.37(0.09) | 2.45E-05 |
|  |  | rs1352170 | 11 | 11062651 | 11p15.3 |  | intergenic | A | -8.75(2.13) | 4.01E-05 | 0.35(0.09) | 5.46E-05 |
|  |  | rs2032414 | 11 | 127547382 | 11q24.3 |  | intergenic | T | 9.35(2.11) | 9.15E-06 | -0.39(0.09) | 5.27E-06 |
|  |  | rs4937864 | 11 | 133395679 | 11q25 |  | intergenic | G | -11.7(2.41) | 1.15E-06 | 0.48(0.10) | 1.19E-06 |
|  |  | rs9512449 | 13 | 26397775 | 13q12.13 |  | intergenic | T | -8.53(2.12) | 5.99E-05 | 0.33(0.09) | 1.12E-04 |
|  |  | rs9634902 | 13 | 62882158 | 13q21.31 |  | intergenic | C | 13.1(3.23) | 4.88E-05 | -0.55(0.13) | 3.34E-05 |
|  |  | rs4885471 | 13 | 76970667 | 13q22.3 |  | intergenic | T | 10.3(2.54) | 5.11E-05 | -0.39(0.10) | 1.35E-04 |
|  |  | rs1551011 | 14 | 84091291 | 14q31.3 |  | intergenic | A | -30.4(6.75) | 6.68E-06 | 1.25(0.27) | 4.11E-06 |
|  |  | rs8025731 | 15 | 52313826 | 15q21.3 | UNC13C | intronic | G | 11.0(2.72) | 5.26E-05 | -0.44(0.11) | 9.01E-05 |
|  |  | rs12594545 | 15 | 64716084 | 15q22.31 |  | intergenic | A | 21.9(5.42) | 5.64E-05 | -0.88(0.22) | 6.52E-05 |
|  |  | rs301627 | 16 | 85209844 | 16q24.1 |  | intergenic | G | 9.30(2.32) | 5.98E-05 | -0.38(0.09) | 6.78E-05 |
|  |  | rs6502615 | 17 | 17552748 | 17p11.2 | RAI1 | intronic | C | 13.9(3.44) | 5.62E-05 | -0.54(0.14) | 1.29E-04 |
|  |  | rs1817630 | 17 | 66050707 | 17q24.3 |  | intergenic | G | -15.8(3.95) | 6.52E-05 | 0.62(0.16) | 1.04E-04 |
|  |  | rs2423402 | 20 | 9597353 | 20p12.2 | PAK7 | intronic | C | -32.5(7.97) | 4.65E-05 | 1.39(0.32) | 1.96E-05 |
|  |  | rs209957 | 20 | 52154502 | 20q13.2 |  | intergenic | G | -15.5(3.47) | 7.63E-06 | 0.63(0.14) | 7.24E-06 |
|  | height | rs309064 | 1 | 99192956 | 1p21.3 | PAP2D | intronic | C | -57.4(13.3) | 1.69E-05 | 0.36(0.08) | 1.49E-05 |
|  |  | rs11805519 | 1 | 65937622 | 1p31.3 |  | intergenic | C | 62.4(12.5) | 5.95E-07 | -0.38(0.08) | 7.88E-07 |
|  |  | rs303928 | 1 | 48264644 | 1p33 |  | intergenic | T | -20.0(5.10) | 9.17E-05 | 0.12(0.03) | 1.44E-04 |
|  |  | rs16862517 | 1 | 19288026 | 1p36.13 | UBR4 | coding | C | 94.9(21.2) | 7.68E-06 | -0.57(0.13) | 1.28E-05 |
|  |  | rs11810181 | 1 | 223075204 | 1q42.12 |  | intergenic | T | -83.0(19.7) | 2.47E-05 | 0.51(0.12) | 2.75E-05 |
|  |  | rs6724823 | 2 | 45080086 | 2p21 |  | intergenic | G | -41.4(8.97) | 3.94E-06 | 0.26(0.06) | 4.75E-06 |
|  |  | rs11128254 | 3 | 72368302 | 3p13 |  | intergenic | C | 22.4(5.50) | 4.53E-05 | -0.14(0.03) | 6.64E-05 |
|  |  | rs1352432 | 4 | 48088191 | 4p12 | SLAIN2 | intronic | G | -36.3(8.99) | 5.59E-05 | 0.22(0.06) | 8.63E-05 |
|  |  | rs920482 | 4 | 67923676 | 4q13.2 |  | intergenic | A | -21.0(5.10) | 3.80E-05 | 0.13(0.03) | 5.16E-05 |
|  |  | rs1993160 | 4 | 82499657 | 4q21.21 |  | intergenic | G | -21.6(5.14) | 2.78E-05 | 0.13(0.03) | 3.36E-05 |
|  |  | rs12509626 | 4 | 100608114 | 4q23 |  | intergenic | C | -30.5(6.81) | 7.54E-06 | 0.19(0.04) | 8.12E-06 |
|  |  | rs17010263 | 4 | 121229157 | 4q27 |  | intergenic | T | 97.3(23.2) | 2.68E-05 | -0.60(0.14) | 3.61E-05 |
|  |  | rs1500801 | 4 | 169904952 | 4q32.3 | PALLD | intronic | A | -38.9(9.73) | 6.53E-05 | 0.24(0.06) | 5.15E-05 |
|  |  | rs6875666 | 5 | 54360391 | 5q11.2 | GZMK | intronic | T | 23.2(5.30) | 1.20E-05 | -0.14(0.03) | 1.36E-05 |
|  |  | rs340048 | 5 | 145183591 | 5q32 | PRELID2 | intronic | G | 23.2(4.97) | 2.94E-06 | -0.14(0.03) | 4.99E-06 |
|  |  | rs4376292 | 5 | 163810798 | 5q34 | BC011998 | non-coding intronic | G | -24.8(6.21) | 6.53E-05 | 0.15(0.04) | 6.95E-05 |
|  |  | rs9296680 | 6 | 52495066 | 6p12.2 | TRAM2 | intronic | C | 83.4(19.7) | 2.32E-05 | -0.51(0.12) | 3.22E-05 |
|  |  | rs13193127 | 6 | 82608516 | 6q14.1 |  | intergenic | C | -20.4(5.04) | 5.09E-05 | 0.13(0.03) | 5.66E-05 |
|  |  | rs940597 | 7 | 67412967 | 7q11.22 |  | intergenic | T | -32.3(7.16) | 6.60E-06 | 0.20(0.04) | 4.36E-06 |
|  |  | rs1320821 | 7 | 144098653 | 7q35 | TPK1 | intronic | G | 25.1(6.01) | 2.99E-05 | -0.15(0.04) | 6.20E-05 |
|  |  | rs2280444 | 8 | 19221383 | 8p21.3 | SH2D4A | coding | A | 49.2(12.6) | 9.97E-05 | -0.30(0.08) | 1.23E-04 |
|  |  | rs11204002 | 8 | 18743939 | 8p22 | PSD3 | intronic | A | 29.4(6.45) | 5.21E-06 | -0.18(0.04) | 5.46E-06 |
|  |  | rs10046679 | 8 | 5929885 | 8p23.2 |  | intergenic | T | 28.9(6.80) | 2.19E-05 | -0.18(0.04) | 1.79E-05 |
|  |  | rs7822836 | 8 | 2011614 | 8p23.3 | MYOM2 | intronic | A | 82.1(20.2) | 4.82E-05 | -0.50(0.13) | 5.61E-05 |
|  |  | rs17735712 | 8 | 85903282 | 8q21.2 | RALYL | intronic | G | 20.8(5.26) | 7.85E-05 | -0.13(0.03) | 9.77E-05 |
|  |  | rs17721181 | 8 | 139371662 | 8q24.23 | FAM135B | intronic | T | 33.2(8.34) | 6.79E-05 | -0.21(0.05) | 7.10E-05 |
|  |  | rs6560281 | 9 | 74288094 | 9q21.13 |  | intergenic | A | -20.0(5.02) | 6.83E-05 | 0.12(0.03) | 7.24E-05 |
|  |  | rs7916609 | 10 | 8694694 | 10p14 |  | intergenic | C | -20.0(5.11) | 9.12E-05 | 0.12(0.03) | 9.57E-05 |
|  |  | rs10822618 | 10 | 67159286 | 10q21.3 |  | intergenic | T | 42.7(10.5) | 5.13E-05 | -0.26(0.07) | 8.80E-05 |
|  |  | rs12354551 | 10 | 85823843 | 10q23.1 |  | intergenic | A | 20.6(5.09) | 5.52E-05 | -0.13(0.03) | 5.35E-05 |
|  |  | rs7948801 | 11 | 98327430 | 11q22.1 |  | intergenic | G | 22.9(5.54) | 3.52E-05 | -0.14(0.03) | 7.93E-05 |
|  |  | rs12426122 | 12 | 18412549 | 12p12.3 | PIK3C2G | intronic | C | 33.2(6.40) | 2.18E-07 | -0.20(0.04) | 4.38E-07 |
|  |  | rs4760818 | 12 | 70665190 | 12q21.1 | TPH2 | intronic | A | -55.7(13.2) | 2.56E-05 | 0.34(0.08) | 3.01E-05 |
|  |  | rs10774942 | 12 | 116522198 | 12q24.22 | KSR2 | intronic | T | 23.9(5.73) | 3.07E-05 | -0.15(0.04) | 2.94E-05 |
|  |  | rs9539079 | 13 | 60724699 | 13q21.31 |  | intergenic | G | -38.5(9.15) | 2.64E-05 | 0.24(0.06) | 3.57E-05 |
|  |  | rs3829405 | 14 | 22172880 | 14q11.2 |  | intergenic | C | 30.6(7.84) | 9.39E-05 | -0.19(0.05) | 1.17E-04 |
|  |  | rs7150130 | 14 | 59897565 | 14q23.1 |  | intergenic | T | 23.7(4.93) | 1.62E-06 | -0.15(0.03) | 1.89E-06 |
|  |  | rs9788522 | 14 | 62752331 | 14q23.2 | RHOJ | intronic | C | 23.2(5.52) | 2.60E-05 | -0.14(0.03) | 2.67E-05 |
|  |  | rs1294489 | 14 | 90019372 | 14q32.11 |  | intergenic | C | -67.1(16.7) | 6.10E-05 | 0.41(0.10) | 6.71E-05 |
|  |  | rs11856206 | 15 | 94396949 | 15q26.2 |  | intergenic | T | 34.7(8.46) | 4.16E-05 | -0.21(0.05) | 6.18E-05 |
|  |  | rs7222349 | 17 | 39660170 | 17q21.31 |  | intergenic | G | -21.9(5.53) | 7.45E-05 | 0.13(0.03) | 1.03E-04 |
|  |  | rs2898883 | 17 | 44837952 | 17q21.33 | PHB | intronic | G | 28.5(6.84) | 3.06E-05 | -0.18(0.04) | 2.24E-05 |
|  |  | rs6081442 | 20 | 18884139 | 20p11.23 |  | intergenic | A | 26.1(5.87) | 8.63E-06 | -0.16(0.04) | 1.15E-05 |
|  |  | rs6117544 | 20 | 698738 | 20p13 | C20orf54 | 5upstream | A | 43.5(10.5) | 3.45E-05 | -0.27(0.07) | 4.67E-05 |
|  |  | rs6029120 | 20 | 38516381 | 20q12 |  | intergenic | T | -32.6(7.65) | 2.06E-05 | 0.20(0.05) | 2.87E-05 |
|  |  | rs237450 | 20 | 47415535 | 20q13.13 | AK055386 | non-coding | G | 29.8(7.24) | 3.97E-05 | -0.18(0.05) | 4.28E-05 |
|  |  | rs5753336 | 22 | 29557373 | 22q12.2 | OSBP2 | intronic | A | 24.5(5.89) | 3.10E-05 | -0.15(0.04) | 3.49E-05 |
|  | weight | rs528077 | 1 | 109867275 | 1p13.3 |  | intergenic | C | 7.36(1.84) | 6.55E-05 | -0.12(0.03) | 7.10E-05 |
|  |  | rs1877719 | 1 | 48600602 | 1p33 | SPATA6 | intronic | A | -7.63(1.80) | 2.21E-05 | 0.12(0.03) | 2.12E-05 |
|  |  | rs2154369 | 1 | 26216497 | 1p36.11 |  | intergenic | G | 7.84(1.86) | 2.52E-05 | -0.12(0.03) | 5.14E-05 |
|  |  | rs360089 | 1 | 224124954 | 1q42.12 | TMEM63A | intronic | T | 9.49(2.40) | 7.61E-05 | -0.14(0.04) | 3.19E-04 |
|  |  | rs41434646 | 2 | 184868757 | 2q32.1 |  | intergenic |  | 20.1(5.05) | 6.78E-05 | -0.29(0.08) | 3.12E-04 |
|  |  | rs6800743 | 3 | 56853987 | 3p14.3 | ARHGEF3 | intronic | T | 10.7(2.47) | 1.51E-05 | -0.15(0.04) | 1.01E-04 |
|  |  | rs9817819 | 3 | 23118522 | 3p24.3 |  | intergenic | C | -7.01(1.69) | 3.22E-05 | 0.11(0.03) | 6.26E-05 |
|  |  | rs10865743 | 3 | 197164 | 3p26.3 |  | intergenic | T | 17.1(3.62) | 2.49E-06 | -0.25(0.06) | 1.40E-05 |
|  |  | rs1488266 | 3 | 113646968 | 3q13.2 |  | intergenic | C | 16.7(3.83) | 1.29E-05 | -0.26(0.06) | 1.75E-05 |
|  |  | rs2068229 | 3 | 143105994 | 3q23 | ATP1B3 | intronic | A | -7.13(1.66) | 1.78E-05 | 0.11(0.03) | 4.29E-05 |
|  |  | rs41401847 | 3 | 145537733 | 3q24 |  | intergenic |  | -17.8(4.25) | 2.90E-05 | 0.27(0.07) | 4.57E-05 |
|  |  | rs2043653 | 4 | 96960809 | 4q22.3 |  | intergenic | T | -11.9(2.85) | 3.10E-05 | 0.17(0.04) | 1.55E-04 |
|  |  | rs12512633 | 4 | 124506365 | 4q28.1 |  | intergenic | T | -7.36(1.78) | 3.56E-05 | 0.11(0.03) | 1.31E-04 |
|  |  | rs13145131 | 4 | 182534660 | 4q34.3 |  | intergenic | T | -7.30(1.85) | 8.25E-05 | 0.11(0.03) | 7.98E-05 |
|  |  | rs17771155 | 5 | 10862094 | 5p15.2 |  | intergenic | G | 17.2(3.82) | 7.07E-06 | -0.25(0.06) | 1.81E-05 |
|  |  | rs11745962 | 5 | 3828647 | 5p15.33 |  | intergenic | G | 30.5(7.57) | 5.53E-05 | -0.46(0.12) | 1.13E-04 |
|  |  | rs13172034 | 5 | 52060154 | 5q11.2 |  | intergenic | T | -8.51(1.89) | 6.97E-06 | 0.13(0.03) | 7.07E-06 |
|  |  | rs6934622 | 6 | 48445351 | 6p12.3 |  | intergenic | T | 10.0(2.50) | 6.16E-05 | -0.15(0.04) | 1.72E-04 |
|  |  | rs9501762 | 6 | 1653855 | 6p25.3 | GMDS | intronic | C | -8.61(1.91) | 6.35E-06 | 0.13(0.03) | 2.28E-05 |
|  |  | rs17645582 | 6 | 82854036 | 6q14.1 |  | intergenic | C | 17.2(4.01) | 1.68E-05 | -0.27(0.06) | 1.63E-05 |
|  |  | rs645106 | 7 | 4156439 | 7p22.2 | SDK1 | intronic | T | 8.51(1.89) | 6.70E-06 | -0.13(0.03) | 1.07E-05 |
|  |  | rs10227746 | 7 | 94325756 | 7q21.3 |  | intergenic | T | -27.7(5.24) | 1.37E-07 | 0.43(0.08) | 1.62E-07 |
|  |  | rs1320821 | 7 | 144098653 | 7q35 | TPK1 | intronic | G | 7.79(2.00) | 9.96E-05 | -0.11(0.03) | 7.33E-04 |
|  |  | rs1949102 | 8 | 60816815 | 8q12.1 |  | intergenic | G | 18.9(4.73) | 6.40E-05 | -0.29(0.07) | 1.02E-04 |
|  |  | rs10102355 | 8 | 102376814 | 8q22.3 |  | intergenic | C | -25.2(6.29) | 6.34E-05 | 0.38(0.10) | 1.39E-04 |
|  |  | rs1516960 | 8 | 129544064 | 8q24.21 |  | intergenic | A | -6.83(1.71) | 6.45E-05 | 0.11(0.03) | 6.53E-05 |
|  |  | rs2889334 | 9 | 36887133 | 9p13.2 | PAX5 | intronic | A | 16.6(3.83) | 1.40E-05 | -0.25(0.06) | 3.25E-05 |
|  |  | rs10975555 | 9 | 6350299 | 9p24.1 |  | intergenic | C | 7.14(1.75) | 4.54E-05 | -0.11(0.03) | 1.01E-04 |
|  |  | rs10760966 | 9 | 105032942 | 9q31.1 | BC035187 | non-coding intronic | G | -6.76(1.63) | 3.42E-05 | 0.10(0.03) | 1.47E-04 |
|  |  | rs11252446 | 10 | 4321581 | 10p15.1 |  | intergenic | T | 21.6(4.84) | 8.34E-06 | -0.33(0.08) | 1.53E-05 |
|  |  | rs10997540 | 10 | 52866483 | 10q11.23 | PRKG1 | intronic | G | 22.9(5.86) | 9.59E-05 | -0.34(0.09) | 2.16E-04 |
|  |  | rs1402363 | 12 | 41616584 | 12q12 |  | intergenic | T | 12.9(3.28) | 8.07E-05 | -0.20(0.05) | 1.21E-04 |
|  |  | rs9317994 | 13 | 23196768 | 13q12.12 |  | intergenic | G | -10.1(2.56) | 8.30E-05 | 0.15(0.04) | 1.48E-04 |
|  |  | rs12583346 | 13 | 100377939 | 13q32.3 |  | intergenic | C | 8.62(2.08) | 3.50E-05 | -0.12(0.03) | 1.71E-04 |
|  |  | rs1956556 | 14 | 59888010 | 14q23.1 |  | intergenic | T | 9.18(1.66) | 3.45E-08 | -0.14(0.03) | 4.61E-08 |
|  |  | rs12594545 | 15 | 64716084 | 15q22.31 | BC016970 | intronic | A | 19.2(4.39) | 1.24E-05 | -0.30(0.07) | 1.58E-05 |
|  |  | rs17209637 | 15 | 77848383 | 15q25.1 |  | intergenic | G | 23.0(5.27) | 1.23E-05 | -0.35(0.08) | 1.91E-05 |
|  |  | rs11652209 | 17 | 66150909 | 17q24.3 |  | intergenic | G | -13.8(3.24) | 1.98E-05 | 0.20(0.05) | 6.16E-05 |
|  |  | rs2288889 | 19 | 43638055 | 19q13.2 | RYR1 | intronic | G | -7.04(1.79) | 8.34E-05 | 0.11(0.03) | 1.41E-04 |
|  |  | rs6093514 | 20 | 39750676 | 20q12 |  | intergenic | A | 8.84(2.15) | 3.87E-05 | -0.12(0.03) | 2.22E-04 |
|  |  | rs6004179 | 22 | 23263069 | 22q11.23 |  | intergenic | C | -12.2(2.89) | 2.48E-05 | 0.19(0.05) | 3.08E-05 |
|  |  | rs6004787 | 22 | 24557767 | 22q12.1 | MYO18B | intronic | C | 15.2(3.71) | 4.38E-05 | -0.22(0.06) | 1.24E-04 |
|  |  | rs6001491 | 22 | 37924686 | 22q13.1 |  | intergenic | C | 8.63(2.12) | 4.75E-05 | -0.14(0.03) | 3.29E-05 |
|  | WHR | rs11586326 | 1 | 102407964 | 1p21.1 |  | intergenic | G | 32.5(6.96) | 3.16E-06 | -36.2(7.87) | 4.31E-06 |
|  |  | rs2043093 | 2 | 73260850 | 2p13.2 |  | intergenic | G | 13.5(3.37) | 6.51E-05 | -15.4(3.82) | 5.60E-05 |
|  |  | rs10169489 | 2 | 40012930 | 2p22.1 | BC043380 | non-coding intronic | G | -13.8(3.25) | 2.00E-05 | 15.4(3.69) | 2.83E-05 |
|  |  | rs7599318 | 2 | 187486596 | 2q32.1 |  | intergenic | A | 13.2(3.39) | 9.72E-05 | -14.5(3.84) | 1.62E-04 |
|  |  | rs11130484 | 3 | 55908333 | 3p14.3 | KIAA0378 | non-coding intronic | A | -13.5(3.30) | 4.47E-05 | 15.3(3.75) | 4.48E-05 |
|  |  | rs16893878 | 5 | 24768486 | 5p14.1 |  | intergenic | C | 18.7(4.74) | 8.24E-05 | -20.9(5.37) | 1.04E-04 |
|  |  | rs3886954 | 6 | 52296568 | 6p12.2 |  | intergenic | C | 17.8(4.47) | 7.19E-05 | -20.8(5.07) | 4.03E-05 |
|  |  | rs9296266 | 6 | 38990614 | 6p21.2 | DNAH8 | intronic | A | -13.4(3.14) | 2.01E-05 | 15.3(3.56) | 1.80E-05 |
|  |  | rs1265238 | 6 | 4444028 | 6p25.1 |  | intergenic | T | -14.3(3.46) | 3.82E-05 | 16.2(3.93) | 3.71E-05 |
|  |  | rs12672946 | 7 | 103212886 | 7q22.1 | RELN | intronic | C | 43.4(10.2) | 2.16E-05 | -50.4(11.7) | 1.51E-05 |
|  |  | rs739617 | 7 | 111298102 | 7q31.1 | DOCK4 | intronic | C | -17.2(4.20) | 4.28E-05 | 19.8(4.78) | 3.37E-05 |
|  |  | rs12343206 | 9 | 126223888 | 9q33.3 |  | intergenic | A | 16.4(3.47) | 2.43E-06 | -19.0(3.94) | 1.54E-06 |
|  |  | rs6482383 | 10 | 24654321 | 10p12.1 | KIAA1217 | intronic | G | -15.1(3.74) | 5.36E-05 | 17.0(4.26) | 6.27E-05 |
|  |  | rs10501680 | 11 | 88117162 | 11q14.3 | GRM5 | intronic | A | -61.8(14.0) | 1.01E-05 | 68.8(15.6) | 1.04E-05 |
|  |  | rs11178246 | 12 | 69145023 | 12q15 |  | intergenic | G | 13.9(3.31) | 2.64E-05 | -16.1(3.77) | 1.87E-05 |
|  |  | rs4380004 | 14 | 104048299 | 14q32.33 | AX746996 | non-coding | T | 19.3(4.86) | 7.40E-05 | -21.7(5.51) | 8.48E-05 |
|  |  | rs2883250 | 16 | 84859632 | 16q24.1 |  | intergenic | G | 17.3(4.39) | 8.01E-05 | -19.9(4.98) | 6.81E-05 |
|  |  | rs8074853 | 17 | 5367839 | 17p13.2 | NLRP1 | intronic | T | 16.2(3.70) | 1.25E-05 | -18.2(4.19) | 1.43E-05 |
|  |  | rs4790044 | 17 | 74960226 | 17q25.3 | HRNBP3 | intronic | G | -14.5(3.4) | 1.80E-05 | 16.3(3.82) | 1.98E-05 |
|  |  | rs2041718 | 18 | 10102756 | 18p11.22 |  | intergenic | A | 16.6(4.02) | 3.75E-05 | -18.4(4.57) | 5.42E-05 |
|  |  | rs2974219 | 19 | 52776841 | 19q13.32 |  | intergenic | G | 37.0(8.98) | 3.92E-05 | -41.5(10.1) | 4.19E-05 |
| DBP | BMI | rs12057453 | 1 | 89114103 | 1p22.2 | GTF2B | intronic | C | -5.82(1.48) | 8.45E-05 | 0.24(0.06) | 4.75E-05 |
|  |  | rs12130333 | 1 | 62964365 | 1p31.3 |  | intergenic | C | -21.4(5.31) | 5.51E-05 | 0.92(0.22) | 2.70E-05 |
|  |  | rs11899367 | 2 | 134510726 | 2q21.2 |  | intergenic | C | -5.51(1.40) | 8.04E-05 | 0.23(0.06) | 6.54E-05 |
|  |  | rs6796871 | 3 | 55861236 | 3p14.3 | MIR3938 | 3downstream | T | -6.38(1.53) | 3.08E-05 | 0.26(0.06) | 2.64E-05 |
|  |  | rs7630022 | 3 | 39090498 | 3p22.2 | WDR48 | intronic | G | 6.34(1.55) | 4.41E-05 | -0.26(0.06) | 3.18E-05 |
|  |  | rs17293627 | 3 | 3664015 | 3p26.2 |  | intergenic | T | -6.36(1.52) | 2.93E-05 | 0.27(0.06) | 1.29E-05 |
|  |  | rs7635013 | 3 | 2050503 | 3p26.3 |  | intergenic | T | 7.61(1.94) | 9.27E-05 | -0.32(0.08) | 5.70E-05 |
|  |  | rs1917779 | 3 | 135082772 | 3q22.1 | RAB6B | intronic | T | -10.1(2.56) | 7.50E-05 | 0.40(0.10) | 1.25E-04 |
|  |  | rs1479852 | 3 | 141576644 | 3q23 | CLSTN2 | intronic | G | -8.45(2.12) | 6.62E-05 | 0.36(0.09) | 3.33E-05 |
|  |  | rs11723162 | 4 | 54330515 | 4q12 |  | intergenic | T | 5.45(1.37) | 7.00E-05 | -0.22(0.06) | 5.65E-05 |
|  |  | rs7697487 | 4 | 148551980 | 4q31.23 |  | intergenic | G | 15.3(3.93) | 9.93E-05 | -0.61(0.16) | 1.13E-04 |
|  |  | rs11100172 | 4 | 159567770 | 4q32.1 |  | intergenic | T | 24.7(6.32) | 9.20E-05 | -0.97(0.25) | 1.18E-04 |
|  |  | rs2584349 | 4 | 187772163 | 4q35.2 | FAT1 | intronic | T | 6.08(1.48) | 3.94E-05 | -0.25(0.06) | 4.43E-05 |
|  |  | rs4401550 | 5 | 101295555 | 5q21.1 |  | intergenic | T | -6.28(1.48) | 2.22E-05 | 0.25(0.06) | 4.83E-05 |
|  |  | rs36070 | 5 | 148397433 | 5q33.1 | SH3TC2 | intronic | T | 11.6(2.38) | 1.05E-06 | -0.47(0.10) | 1.50E-06 |
|  |  | rs9406226 | 6 | 8890689 | 6p24.3 |  | intergenic | G | 5.40(1.37) | 8.61E-05 | -0.22(0.06) | 9.67E-05 |
|  |  | rs6945638 | 7 | 55909567 | 7p11.2 |  | intergenic | C | -22.2(5.47) | 4.96E-05 | 0.88(0.22) | 8.88E-05 |
|  |  | rs6960015 | 7 | 51802770 | 7p12.1 |  | intergenic | T | -23.9(5.50) | 1.41E-05 | 0.98(0.22) | 1.23E-05 |
|  |  | rs645106 | 7 | 4156439 | 7p22.2 | SDK1 | intronic | T | 6.26(1.57) | 6.75E-05 | -0.25(0.06) | 1.01E-04 |
|  |  | rs1365016 | 8 | 25595747 | 8p21.2 |  | intergenic | C | -6.03(1.54) | 8.60E-05 | 0.24(0.06) | 9.32E-05 |
|  |  | rs7016467 | 8 | 13063130 | 8p22 | DLC1 | intronic | G | -5.66(1.38) | 4.32E-05 | 0.23(0.06) | 5.30E-05 |
|  |  | rs2465985 | 8 | 67516280 | 8q13.1 | ADHFE1 | intronic | C | -5.89(1.45) | 4.96E-05 | 0.25(0.06) | 2.47E-05 |
|  |  | rs7017987 | 8 | 87742749 | 8q21.3 | CNGB3 | intronic | A | -9.55(2.35) | 4.74E-05 | 0.38(0.10) | 7.51E-05 |
|  |  | rs2153460 | 9 | 84765998 | 9q21.32 |  | intergenic | T | -13.6(3.48) | 9.71E-05 | 0.54(0.14) | 1.33E-04 |
|  |  | rs1339761 | 9 | 109739598 | 9q31.2 |  | intergenic | G | 21.7(5.57) | 9.52E-05 | -0.87(0.23) | 1.40E-04 |
|  |  | rs4937864 | 11 | 133395679 | 11q25 |  | intergenic | G | -7.14(1.58) | 6.47E-06 | 0.29(0.06) | 4.94E-06 |
|  |  | rs3913144 | 12 | 23045563 | 12p12.1 |  | intergenic | C | -5.66(1.44) | 8.61E-05 | 0.23(0.06) | 7.21E-05 |
|  |  | rs6538403 | 12 | 92197630 | 12q22 | LOC643339 | non-coding intronic | G | -8.32(2.07) | 5.75E-05 | 0.34(0.08) | 5.21E-05 |
|  |  | rs35338 | 12 | 114099800 | 12q24.21 |  | intergenic | A | -11.8(2.45) | 1.36E-06 | 0.46(0.10) | 3.11E-06 |
|  |  | rs4942930 | 13 | 50071281 | 13q14.3 |  | intergenic | C | -5.49(1.41) | 9.47E-05 | 0.23(0.06) | 5.68E-05 |
|  |  | rs6492104 | 13 | 107768237 | 13q33.3 |  | intergenic | T | -13.8(3.44) | 6.09E-05 | 0.57(0.14) | 4.05E-05 |
|  |  | rs4898839 | 14 | 54031482 | 14q22.2 |  | intergenic | G | 6.33(1.61) | 8.42E-05 | -0.26(0.07) | 7.70E-05 |
|  |  | rs1551011 | 14 | 84091291 | 14q31.3 |  | intergenic | A | -19.1(4.43) | 1.62E-05 | 0.81(0.18) | 5.87E-06 |
|  |  | rs4073321 | 15 | 76909653 | 15q25.1 |  | intergenic | T | 5.78(1.42) | 4.65E-05 | -0.23(0.06) | 7.89E-05 |
|  |  | rs12444219 | 16 | 23057528 | 16p12.1 | USP31 | intronic | T | 9.56(2.02) | 2.27E-06 | -0.38(0.08) | 4.91E-06 |
|  |  | rs6497657 | 16 | 23108599 | 16p12.1 | SCNN1G | intronic | T | 8.38(2.06) | 4.85E-05 | -0.33(0.08) | 7.95E-05 |
|  |  | rs13329823 | 16 | 12694285 | 16p13.12 | CPPED1 | intronic | T | 16.7(4.21) | 7.57E-05 | -0.66(0.17) | 1.11E-04 |
|  |  | rs2052779 | 18 | 10365022 | 18p11.22 |  | intergenic | T | -6.37(1.59) | 6.36E-05 | 0.26(0.06) | 6.16E-05 |
|  |  | rs1521797 | 18 | 30284759 | 18q12.1 |  | intergenic | T | -5.75(1.45) | 7.74E-05 | 0.23(0.06) | 1.22E-04 |
|  |  | rs8110950 | 19 | 61589101 | 19q13.43 | ZNF582 | intronic | C | -7.01(1.60) | 1.21E-05 | 0.28(0.07) | 1.22E-05 |
|  |  | rs968746 | 20 | 9444426 | 20p12.2 | LAMP5 | intronic | T | 8.22(2.11) | 9.53E-05 | -0.31(0.09) | 2.21E-04 |
|  |  | rs1015773 | 20 | 39728021 | 20q12 |  | intergenic | C | -5.65(1.42) | 7.29E-05 | 0.22(0.06) | 1.88E-04 |
|  |  | rs209954 | 20 | 52156350 | 20q13.2 |  | intergenic | T | -10.1(2.27) | 9.97E-06 | 0.41(0.09) | 8.88E-06 |
|  | height | rs309064 | 1 | 99192956 | 1p21.3 | PAP2D | intronic | C | -37.1(8.85) | 2.82E-05 | 0.23(0.06) | 2.21E-05 |
|  |  | rs11805519 | 1 | 65937622 | 1p31.3 |  | intergenic | C | 35.4(8.30) | 2.06E-05 | -0.22(0.05) | 2.38E-05 |
|  |  | rs6587829 | 1 | 58775113 | 1p32.1 | DAB1 | intronic | G | 20.8(4.96) | 2.79E-05 | -0.13(0.03) | 3.60E-05 |
|  |  | rs6691050 | 1 | 48279865 | 1p33 |  | intergenic | G | 13.5(3.34) | 5.31E-05 | -0.08(0.02) | 7.71E-05 |
|  |  | rs16862556 | 1 | 19324488 | 1p36.13 | UBR4 | intronic | G | 57.6(14.3) | 5.43E-05 | -0.35(0.09) | 6.91E-05 |
|  |  | rs1527186 | 2 | 78125305 | 2p12 | BC030125 | non-coding intronic | A | 20.6(4.83) | 2.03E-05 | -0.13(0.03) | 3.07E-05 |
|  |  | rs6724823 | 2 | 45080086 | 2p21 |  | intergenic | G | -25.1(5.96) | 2.50E-05 | 0.16(0.04) | 2.24E-05 |
|  |  | rs10511127 | 3 | 78677401 | 3p12.3 |  | intergenic | C | 28.4(6.87) | 3.62E-05 | -0.18(0.04) | 4.21E-05 |
|  |  | rs4547722 | 3 | 20209528 | 3p24.3 |  | intergenic | C | -17.8(4.53) | 8.88E-05 | 0.11(0.03) | 7.72E-05 |
|  |  | rs718130 | 3 | 136191967 | 3q22.2 | EPHB1 | intronic | A | -61.0(15.4) | 7.93E-05 | 0.39(0.10) | 4.61E-05 |
|  |  | rs1352437 | 4 | 84974220 | 4q21.23 | BC005018 | non-coding intronic | C | -16.1(3.77) | 1.84E-05 | 0.10(0.02) | 2.05E-05 |
|  |  | rs2869432 | 4 | 87492344 | 4q21.3 | MAPK10 | intronic | C | 17.0(4.13) | 3.95E-05 | -0.10(0.03) | 4.41E-05 |
|  |  | rs12509626 | 4 | 100608114 | 4q23 |  | intergenic | C | -18.7(4.53) | 3.55E-05 | 0.12(0.03) | 3.01E-05 |
|  |  | rs6532945 | 4 | 102702806 | 4q24 | BANK1 | intronic | T | -15.4(3.51) | 1.17E-05 | 0.10(0.02) | 1.08E-05 |
|  |  | rs6553938 | 4 | 177389416 | 4q34.2 | ASB5 | intronic | T | -19.9(3.97) | 5.38E-07 | 0.12(0.02) | 7.05E-07 |
|  |  | rs12498570 | 4 | 178572244 | 4q34.3 |  | intergenic | T | -29.3(7.25) | 5.28E-05 | 0.18(0.05) | 5.05E-05 |
|  |  | rs340048 | 5 | 145183591 | 5q32 | PRELID2 | intronic | G | 14.1(3.30) | 1.88E-05 | -0.09(0.02) | 2.87E-05 |
|  |  | rs9404263 | 6 | 96681392 | 6q16.1 | FUT9 | intronic | G | 13.9(3.40) | 4.45E-05 | -0.09(0.02) | 5.17E-05 |
|  |  | rs7806797 | 7 | 141183706 | 7q34 | LOC136242 | intronic | C | -15.4(3.73) | 3.44E-05 | 0.10(0.02) | 3.53E-05 |
|  |  | rs3213197 | 8 | 31041668 | 8p12 | WRN | intronic | A | 51.9(13.1) | 7.53E-05 | -0.32(0.08) | 8.87E-05 |
|  |  | rs2614063 | 8 | 28213320 | 8p21.1 |  | intergenic | C | 17.6(4.50) | 9.80E-05 | -0.11(0.03) | 9.76E-05 |
|  |  | rs560960 | 9 | 18734405 | 9p22.1 | ADAMTSL1 | intronic | T | 13.4(3.35) | 6.03E-05 | -0.08(0.02) | 6.67E-05 |
|  |  | rs2431636 | 10 | 12915291 | 10p13 | BC073155 | 5upstream | G | 34.4(7.84) | 1.20E-05 | -0.21(0.05) | 1.22E-05 |
|  |  | rs2928391 | 10 | 49667278 | 10q11.22 | WDFY4 | intronic | A | -16.2(3.74) | 1.53E-05 | 0.10(0.02) | 1.30E-05 |
|  |  | rs10996460 | 10 | 66909605 | 10q21.3 |  | intergenic | T | -16.7(4.07) | 3.93E-05 | 0.10(0.03) | 6.12E-05 |
|  |  | rs16933541 | 10 | 77866180 | 10q22.3 | C10orf11 | intronic | T | -21.2(5.46) | 9.94E-05 | 0.13(0.03) | 1.08E-04 |
|  |  | rs12354551 | 10 | 85823843 | 10q23.1 |  | intergenic | A | 13.4(3.38) | 7.80E-05 | -0.08(0.02) | 7.01E-05 |
|  |  | rs572311 | 11 | 125574842 | 11q24.2 |  | intergenic | G | 19.6(4.89) | 5.87E-05 | -0.12(0.03) | 6.03E-05 |
|  |  | rs11044057 | 12 | 18426133 | 12p12.3 | PIK3C2G | intronic | C | 19.4(4.26) | 5.05E-06 | -0.12(0.03) | 6.40E-06 |
|  |  | rs1486346 | 12 | 37418220 | 12q12 | CPNE8 | intronic | T | 21.7(5.08) | 2.03E-05 | -0.13(0.03) | 2.56E-05 |
|  |  | rs2805962 | 13 | 62451877 | 13q21.31 |  | intergenic | G | 55.2(12.8) | 1.57E-05 | -0.35(0.08) | 1.47E-05 |
|  |  | rs17064038 | 13 | 74729964 | 13q22.2 |  | intergenic | C | 18.5(4.72) | 9.00E-05 | -0.12(0.03) | 7.36E-05 |
|  |  | rs1956665 | 14 | 62508786 | 14q23.2 | KCNH5 | intronic | G | 17.2(4.35) | 8.20E-05 | -0.11(0.03) | 1.05E-04 |
|  |  | rs8021155 | 14 | 85080010 | 14q31.3 | FLRT2 | intronic | C | 26.9(6.29) | 1.88E-05 | -0.17(0.04) | 1.92E-05 |
|  |  | rs4591171 | 16 | 74858358 | 16q23.1 |  | intergenic | T | -38.5(9.38) | 4.05E-05 | 0.24(0.06) | 4.40E-05 |
|  |  | rs254321 | 16 | 82308939 | 16q23.3 | CDH13 | intronic | C | -15.2(3.67) | 3.23E-05 | 0.09(0.02) | 3.29E-05 |
|  |  | rs9897342 | 17 | 24548627 | 17q11.2 |  | intergenic | C | 13.6(3.41) | 6.37E-05 | -0.09(0.02) | 5.16E-05 |
|  |  | rs11869101 | 17 | 43923012 | 17q21.32 |  | intergenic | T | -15.4(3.89) | 7.23E-05 | 0.09(0.02) | 9.14E-05 |
|  |  | rs2898883 | 17 | 44837952 | 17q21.33 | PHB | intronic | G | 18.3(4.55) | 5.91E-05 | -0.11(0.03) | 6.69E-05 |
|  |  | rs6045692 | 20 | 18849412 | 20p11.23 |  | intergenic | C | 15.0(3.75) | 6.13E-05 | -0.09(0.02) | 7.22E-05 |
|  |  | rs6072215 | 20 | 39030014 | 20q12 |  | intergenic | C | -16.0(3.93) | 4.78E-05 | 0.10(0.02) | 4.65E-05 |
|  | weight | rs1528685 | 1 | 106107627 | 1p21.1 |  | intergenic | C | -4.57(1.10) | 3.37E-05 | 0.07(0.02) | 2.93E-05 |
|  |  | rs17163470 | 1 | 26223969 | 1p36.11 | EXTL1 | intronic | T | 4.93(1.19) | 3.67E-05 | -0.07(0.02) | 9.01E-05 |
|  |  | rs12080175 | 1 | 9039330 | 1p36.23 | SLC2A5 | intronic | T | -4.31(1.10) | 8.33E-05 | 0.06(0.02) | 1.60E-04 |
|  |  | rs6685742 | 1 | 169941397 | 1q24.3 | VAMP4 | non-coding intronic | G | -4.79(1.14) | 2.51E-05 | 0.07(0.02) | 5.63E-05 |
|  |  | rs1935660 | 1 | 187509749 | 1q31.1 |  | intergenic | A | 4.52(1.07) | 2.49E-05 | -0.07(0.02) | 3.91E-05 |
|  |  | rs17013628 | 1 | 207139563 | 1q32.2 |  | intergenic | C | -5.30(1.21) | 1.23E-05 | 0.09(0.02) | 5.06E-06 |
|  |  | rs1511049 | 1 | 212853060 | 1q41 | CENPF | intronic | T | 6.61(1.61) | 4.17E-05 | -0.11(0.03) | 2.35E-05 |
|  |  | rs4849144 | 2 | 113516520 | 2q13 | IL1F8 | intronic | A | 10.0(2.38) | 2.48E-05 | -0.15(0.04) | 3.73E-05 |
|  |  | rs2321552 | 2 | 134509341 | 2q21.2 |  | intergenic | T | -5.13(1.24) | 3.42E-05 | 0.08(0.02) | 3.81E-05 |
|  |  | rs1424659 | 2 | 170794073 | 2q31.1 | MYO3B | intronic | T | -4.56(1.16) | 8.83E-05 | 0.08(0.02) | 3.52E-05 |
|  |  | rs2360096 | 2 | 195076316 | 2q32.3 |  | intergenic | G | -4.63(1.13) | 4.49E-05 | 0.07(0.02) | 1.19E-04 |
|  |  | rs10865743 | 3 | 197164 | 3p26.3 |  | intergenic | T | 9.59(2.38) | 5.64E-05 | -0.14(0.04) | 1.75E-04 |
|  |  | rs1488266 | 3 | 113646968 | 3q13.2 |  | intergenic | C | 10.4(2.52) | 3.52E-05 | -0.16(0.04) | 3.84E-05 |
|  |  | rs11932474 | 4 | 47692010 | 4p12 | CNGA1 | intronic | A | 12.4(3.17) | 9.11E-05 | -0.20(0.05) | 4.30E-05 |
|  |  | rs4698493 | 4 | 16158546 | 4p15.32 | LDB2 | intronic | C | 5.07(1.29) | 8.93E-05 | -0.08(0.02) | 9.47E-05 |
|  |  | rs12650724 | 4 | 11865523 | 4p15.33 |  | intergenic | A | -4.61(1.18) | 9.25E-05 | 0.07(0.02) | 9.50E-05 |
|  |  | rs12499621 | 4 | 100603690 | 4q23 |  | intergenic | T | -5.96(1.48) | 5.91E-05 | 0.10(0.02) | 3.89E-05 |
|  |  | rs13147524 | 4 | 147547933 | 4q31.22 | SLC10A7 | intronic | G | -5.75(1.38) | 2.94E-05 | 0.09(0.02) | 2.55E-05 |
|  |  | rs1542384 | 4 | 159664766 | 4q32.1 | RXFP1 | intronic | T | 7.25(1.84) | 8.35E-05 | -0.11(0.03) | 6.79E-05 |
|  |  | rs2895784 | 5 | 101228131 | 5q21.1 |  | intergenic | A | -4.59(1.14) | 5.62E-05 | 0.07(0.02) | 1.72E-04 |
|  |  | rs9501762 | 6 | 1653855 | 6p25.3 | GMDS | intronic | C | -5.04(1.25) | 5.98E-05 | 0.07(0.02) | 1.44E-04 |
|  |  | rs7744226 | 6 | 127939904 | 6q22.33 | C6orf58 | 5upstream | C | -8.05(2.07) | 9.81E-05 | 0.12(0.03) | 1.76E-04 |
|  |  | rs12529283 | 6 | 162535596 | 6q26 | parkin | non-coding intronic | G | 5.06(1.29) | 8.51E-05 | -0.08(0.02) | 1.77E-04 |
|  |  | rs17829881 | 7 | 52575268 | 7p12.1 |  | intergenic | A | 12.0(3.04) | 8.07E-05 | -0.18(0.05) | 1.62E-04 |
|  |  | rs935567 | 7 | 4172983 | 7p22.2 | SDK1 | intronic | A | 5.75(1.24) | 3.89E-06 | -0.09(0.02) | 5.25E-06 |
|  |  | rs10227746 | 7 | 94325756 | 7q21.3 |  | intergenic | T | -14.5(3.45) | 2.62E-05 | 0.24(0.05) | 1.27E-05 |
|  |  | rs1320821 | 7 | 144098653 | 7q35 | TPK1 | intronic | G | 5.87(1.32) | 8.18E-06 | -0.08(0.02) | 5.04E-05 |
|  |  | rs7016467 | 8 | 13063130 | 8p22 | DLC1 | intronic | G | -5.74(1.11) | 2.50E-07 | 0.09(0.02) | 2.62E-07 |
|  |  | rs2465985 | 8 | 67516280 | 8q13.1 | ADHFE1 | intronic | C | -5.16(1.16) | 9.52E-06 | 0.09(0.02) | 2.95E-06 |
|  |  | rs4243867 | 8 | 138320099 | 8q24.23 |  | intergenic | C | -10.6(2.63) | 5.29E-05 | 0.16(0.04) | 6.61E-05 |
|  |  | rs6992207 | 8 | 144269926 | 8q24.3 |  | intergenic | C | -4.84(1.24) | 9.74E-05 | 0.08(0.02) | 3.76E-05 |
|  |  | rs7466692 | 9 | 36887799 | 9p13.2 | PAX5 | intronic | C | 11.4(2.57) | 8.48E-06 | -0.17(0.04) | 2.18E-05 |
|  |  | rs2448544 | 10 | 49678293 | 10q11.22 | WDFY4 | intronic | G | -5.04(1.24) | 5.15E-05 | 0.08(0.02) | 2.80E-05 |
|  |  | rs10887395 | 10 | 86651943 | 10q23.1 |  | intergenic | A | -4.45(1.11) | 6.42E-05 | 0.07(0.02) | 5.86E-05 |
|  |  | rs3750562 | 10 | 99416405 | 10q24.2 | PI4K2A | intronic | G | 8.64(1.97) | 1.18E-05 | -0.14(0.03) | 6.72E-06 |
|  |  | rs8181552 | 11 | 36267636 | 11p13 | COMMD9 | 5upstream | G | 5.71(1.45) | 8.31E-05 | -0.09(0.02) | 4.92E-05 |
|  |  | rs638433 | 11 | 125685580 | 11q24.2 | DCPS | intronic | T | 6.80(1.66) | 4.21E-05 | -0.11(0.03) | 3.28E-05 |
|  |  | rs10894279 | 11 | 130276978 | 11q24.3 | SNX19 | intronic | T | 4.83(1.16) | 3.34E-05 | -0.07(0.02) | 1.20E-04 |
|  |  | rs2120576 | 12 | 91754320 | 12q22 | EEA1 | intronic | T | 4.60(1.09) | 2.64E-05 | -0.07(0.02) | 2.34E-05 |
|  |  | rs907077 | 12 | 114129650 | 12q24.21 |  | intergenic | T | -11.3(2.87) | 8.80E-05 | 0.17(0.04) | 1.95E-04 |
|  |  | rs2942379 | 12 | 120637513 | 12q24.31 | TMEM120B | intronic | C | -4.30(1.10) | 9.10E-05 | 0.07(0.02) | 1.30E-04 |
|  |  | rs7323227 | 13 | 61354554 | 13q21.31 |  | intergenic | T | -13.9(3.42) | 4.91E-05 | 0.22(0.05) | 4.34E-05 |
|  |  | rs9531050 | 13 | 79821830 | 13q31.1 |  | intergenic | G | -5.08(1.27) | 6.09E-05 | 0.08(0.02) | 7.34E-05 |
|  |  | rs17092838 | 14 | 50023880 | 14q22.1 | MAP4K5 | intronic | A | 9.05(2.07) | 1.26E-05 | -0.13(0.03) | 3.11E-05 |
|  |  | rs10131255 | 14 | 62515815 | 14q23.2 | KCNH5 | intronic | G | 6.25(1.56) | 6.31E-05 | -0.09(0.02) | 1.18E-04 |
|  |  | rs17102601 | 14 | 64859324 | 14q23.3 |  | intergenic | A | -9.50(2.14) | 9.17E-06 | 0.14(0.03) | 2.42E-05 |
|  |  | rs12599183 | 16 | 20186115 | 16p12.3 |  | intergenic | C | 6.98(1.77) | 8.38E-05 | -0.11(0.03) | 3.95E-05 |
|  | WHR | rs11811488 | 1 | 102255799 | 1p21.1 |  | intergenic | C | 20.0(4.84) | 3.47E-05 | -22.9(5.46) | 2.88E-05 |
|  |  | rs10917874 | 1 | 162154706 | 1q23.3 |  | intergenic | A | 36.3(8.27) | 1.15E-05 | -40.3(9.36) | 1.68E-05 |
|  |  | rs10916425 | 1 | 227330239 | 1q42.13 |  | intergenic | C | 8.70(2.10) | 3.49E-05 | -10.0(2.38) | 2.78E-05 |
|  |  | rs7512173 | 1 | 230075529 | 1q42.2 | DISC1 | intronic | C | -8.73(2.13) | 4.13E-05 | 10.2(2.42) | 2.73E-05 |
|  |  | rs11897469 | 2 | 29496575 | 2p23.2 | ALK | intronic | A | -29.1(7.27) | 6.19E-05 | 32.6(8.21) | 7.27E-05 |
|  |  | rs12611650 | 2 | 6312037 | 2p25.2 |  | intergenic | T | 8.62(2.12) | 4.61E-05 | -9.73(2.40) | 5.18E-05 |
|  |  | rs10185508 | 2 | 132048916 | 2q21.1 |  | intergenic | C | -8.88(2.11) | 2.62E-05 | 10.0(2.40) | 3.15E-05 |
|  |  | rs2376344 | 2 | 140003822 | 2q22.1 |  | intergenic | C | 10.9(2.55) | 2.13E-05 | -12.3(2.90) | 2.07E-05 |
|  |  | rs10182631 | 2 | 190208204 | 2q32.2 |  | intergenic | C | 18.9(4.54) | 3.05E-05 | -21.3(5.11) | 3.04E-05 |
|  |  | rs995842 | 2 | 220569837 | 2q35 |  | intergenic | C | -9.08(2.11) | 1.66E-05 | 10.3(2.39) | 1.81E-05 |
|  |  | rs4281890 | 2 | 226992450 | 2q36.3 |  | intergenic | A | -9.53(2.35) | 5.11E-05 | 10.6(2.67) | 7.23E-05 |
|  |  | rs13093307 | 3 | 70465547 | 3p14.1 |  | intergenic | C | -9.63(2.43) | 7.35E-05 | 11.3(2.75) | 3.83E-05 |
|  |  | rs12634217 | 3 | 44903488 | 3p21.31 | TGM4 | intronic | T | 23.3(5.68) | 4.06E-05 | -26.7(6.43) | 3.32E-05 |
|  |  | rs6550428 | 3 | 36631238 | 3p22.2 |  | intergenic | A | -9.81(2.45) | 6.20E-05 | 11.2(2.78) | 5.35E-05 |
|  |  | rs4684782 | 3 | 11493755 | 3p25.3 | ATG7 | intronic | T | -8.74(2.14) | 4.36E-05 | 10.2(2.43) | 2.69E-05 |
|  |  | rs41401847 | 3 | 145537733 | 3q24 |  | intergenic | G | -9.00(2.24) | 6.02E-05 | 10.1(2.55) | 7.32E-05 |
|  |  | rs6783043 | 3 | 191067902 | 3q28 | TP63 | intronic | A | 9.32(2.10) | 9.07E-06 | -10.3(2.38) | 1.46E-05 |
|  |  | rs16893878 | 5 | 24768486 | 5p14.1 |  | intergenic | C | 12.3(3.13) | 9.21E-05 | -13.8(3.55) | 9.77E-05 |
|  |  | rs6450430 | 5 | 57018072 | 5q11.2 |  | intergenic | C | 11.4(2.73) | 2.90E-05 | -12.8(3.10) | 3.76E-05 |
|  |  | rs6943592 | 7 | 41360708 | 7p14.1 |  | intergenic | G | 45.5(9.50) | 1.71E-06 | -51.1(10.68) | 1.72E-06 |
|  |  | rs7805959 | 7 | 126098318 | 7q31.33 | GRM8 | intronic | C | -12.5(3.21) | 9.84E-05 | 14.2(3.65) | 1.06E-04 |
|  |  | rs10458275 | 7 | 139968031 | 7q34 | DENND2A | intronic | A | -13.4(3.42) | 8.65E-05 | 15.3(3.89) | 7.99E-05 |
|  |  | rs2383778 | 9 | 28743170 | 9p21.1 |  | intergenic | C | -8.98(2.24) | 6.35E-05 | 10.6(2.55) | 3.01E-05 |
|  |  | rs10982866 | 9 | 117476374 | 9q33.1 |  | intergenic | C | -8.57(2.10) | 4.54E-05 | 9.32(2.38) | 9.25E-05 |
|  |  | rs12346358 | 9 | 126213912 | 9q33.3 | PSMB7 | intronic | G | -9.08(2.13) | 1.95E-05 | 10.6(2.41) | 1.23E-05 |
|  |  | rs4335438 | 10 | 18836831 | 10p12.33 | CACNB2 | intronic | T | 8.66(2.11) | 4.08E-05 | -9.63(2.39) | 5.70E-05 |
|  |  | rs10509008 | 10 | 55709342 | 10q21.1 | PCDH15 | intronic | T | -8.90(2.18) | 4.73E-05 | 10.3(2.48) | 3.66E-05 |
|  |  | rs826443 | 10 | 72045548 | 10q22.1 |  | intergenic | T | -13.2(3.22) | 4.41E-05 | 15.1(3.66) | 3.82E-05 |
|  |  | rs10838567 | 11 | 46092919 | 11p11.2 | PHF21A | intronic | G | -8.78(2.18) | 5.65E-05 | 10.0(2.48) | 5.18E-05 |
|  |  | rs11025836 | 11 | 20936613 | 11p15.1 | NELL1 | intronic | G | -8.73(2.17) | 5.61E-05 | 10.3(2.46) | 2.87E-05 |
|  |  | rs4243950 | 11 | 9623048 | 11p15.4 |  | intergenic | A | 9.37(2.25) | 3.06E-05 | -11.1(2.55) | 1.44E-05 |
|  |  | rs7938536 | 11 | 2738572 | 11p15.5 | KCNQ1 | intronic | A | -12.6(2.91) | 1.59E-05 | 14.1(3.30) | 2.09E-05 |
|  |  | rs349081 | 11 | 84489038 | 11q14.1 | DLG2 | intronic | A | 11.1(2.82) | 7.57E-05 | -12.7(3.20) | 7.24E-05 |
|  |  | rs4899880 | 14 | 85289931 | 14q31.3 |  | intergenic | G | -9.56(2.30) | 3.29E-05 | 11.0(2.61) | 2.54E-05 |
|  |  | rs6564760 | 16 | 79042599 | 16q23.2 |  | intergenic | G | 8.72(2.13) | 4.25E-05 | -9.81(2.42) | 5.15E-05 |
|  |  | rs2883250 | 16 | 84859632 | 16q24.1 |  | intergenic | G | 13.9(2.90) | 1.52E-06 | -15.8(3.29) | 1.64E-06 |
|  |  | rs2041718 | 18 | 10102756 | 18p11.22 |  | intergenic | A | 10.7(2.65) | 5.24E-05 | -12.0(3.02) | 7.50E-05 |
|  |  | rs8089138 | 18 | 19777061 | 18q11.2 | LAMA3 | intronic | C | -9.18(2.15) | 1.94E-05 | 10.3(2.44) | 2.59E-05 |
|  |  | rs470469 | 18 | 72800135 | 18q23 | ZNF236 | intronic | C | -8.75(2.07) | 2.51E-05 | 10.1(2.35) | 1.95E-05 |
|  |  | rs743296 | 21 | 38566716 | 21q22.13 | KCNJ15 | intronic | G | 8.86(2.23) | 7.22E-05 | -10.2(2.54) | 5.87E-05 |
| SBP, systolic blood pressure; DBP, diastolic blood pressure; CHR, chromosome; MA, minor allele; BMI, body mass index; WHR, waist-hip ratio;  ^1^ Beta and s.e. for SNP main effect, ^2^ *P* value for SNP main effect, ^3^ Beta and s.e. for interactional effect, ^4^ *P* value for interactional effect; CHR, chromosome; MA, minor allele; | | | | | | | | | | | | |

| **Table S4** Results of combined meta-analyses for selected 6 SNPs | | |  |  |  |  |  | | | | |  | |  |  |  | |  | | |  | |  |  | |  | |  | |  |  | |  | |  |
| --- | --- | --- | --- | --- | --- | --- | --- | --- | --- | --- | --- | --- | --- | --- | --- | --- | --- | --- | --- | --- | --- | --- | --- | --- | --- | --- | --- | --- | --- | --- | --- | --- | --- | --- | --- |
| **Trait** | **Interaction** | **SNP; CHR; Location(bp); Cytoband** | **Nearby**  **genes** | **Function** | **MA** | **effect type** | **Discovery (n=7,486)** | | |  | **Replication 1 (n=3,703)** | | | | | | | |  | **Replication 2 (n=841)** | | | | | | |  | | **Combined (n=12,030)** | | | | | | |
|  |  |  |  |  |  |  | **MAF** | **Beta(se)** | ***P*** |  | **MAF** | | **Beta(se)** | | | | ***P*** | |  | **MAF** | | **Beta(se)** | | | ***P*** | |  | | **Beta(se)** | | | ***P*** | | ***P_het_*(Q)** | |
| SBP | BMI | rs13390641; 2; 103402865; 2q12.1 | TMEM182 |  | A | SNP main | 0.11 | -13.9 (3.35) | 3.51E-05 |  | 0.10 | | -11.6 (4.35) | | | | 7.73E-03 | |  | 0.10 | | -32.1 (12.5) | | | 0.010 | |  | | -14.4 (2.62) | | | 3.83E-08 | | 0.47 (2.52) | |
|  |  |  |  |  |  | Interaction |  | 0.56 (0.14) | 3.80E-05 |  |  |  | 0.47 (0.18) | | | | 9.39E-03 | |  |  |  | 1.35 (0.54) | | | 0.012 | |  | | 0.59 (0.11) | | | 5.28E-08 | | 0.48 (2.49) | |
|  | Height | rs9539079; 13; 60724699; 13q21.31 | PCDH20 |  | C | SNP main | 0.08 | -38.5 (9.15) | 2.64E-05 |  | 0.10 | | -21.1 (10.2) | | | | 3.86E-02 | |  | 0.10 | | 20.4 (31.2) | | | 0.514 | |  | | -29.0 (6.86) | | | 2.32E-05 | | 0.23 (4.29) | |
|  |  |  |  |  |  | Interaction |  | 0.24 (0.06) | 3.57E-05 |  |  |  | 0.13 (0.06) | | | | 4.39E-02 | |  |  |  | -0.11 (0.19) | | | 0.577 | |  | | 0.18 (0.04) | | | 2.92E-05 | | 0.26 (4.00) | |
|  | Weight | *rs10760966; 9; 105032942; 9q31.1 | BC035187 | non-coding intronic | A | SNP main | 0.47 | -6.76 (1.63) | 3.42E-05 |  | 0.46 | | -4.47 (2.04) | | | | 2.85E-02 | |  | 0.46 | | 4.15 (7.56) | | | 0.583 | |  | | -5.69 (1.26) | | | 6.88E-06 | | 0.29 (2.47) | |
|  |  |  |  |  |  | Interaction |  | 0.10 (0.03) | 1.47E-04 |  |  |  | 0.07 (0.03) | | | | 3.05E-02 | |  |  |  | -0.05 (0.13) | | | 0.693 | |  | | 0.08 (0.02) | | | 2.71E-05 | | 0.47 (1.52) | |
| DBP | BMI | rs6492104; 13; 107768237; 13q33.3 | TNFSF13B, MYO16 |  | C | SNP main | 0.04 | -13.8 (3.44) | 6.09E-05 |  | 0.04 | | -5.73 (2.26) | | | | 1.14E-02 | |  | 0.02 | | -11.5 (17.5) | | | 0.513 | |  | | -11.2 (2.56) | | | 1.35E-05 | | 0.27 (3.90) | |
|  |  |  |  |  |  | Interaction |  | 0.57 (0.14) | 4.05E-05 |  |  |  | 0.24 (0.09) | | | | 1.05E-02 | |  |  |  | 0.48 (0.78) | | | 0.540 | |  | | 0.46 (0.11) | | | 1.33E-05 | | 0.25 (4.07) | |
|  | Height | rs309064; 1; 99192956; 1p21.3 | PAP2D | intronic | C | SNP main | 0.03 | -37.1 (8.85) | 2.82E-05 |  | 0.04 | | -14.2 (6.60) | | | | 3.13E-02 | |  | 0.05 | | -26.2 (30.2) | | | 0.387 | |  | | -29.3 (6.24) | | | 2.69E-06 | | 0.15 (5.37) | |
|  |  |  |  |  |  | Interaction |  | 0.23 (0.06) | 2.21E-05 |  |  |  | 0.09 (0.04) | | | | 2.34E-02 | |  |  |  | 0.17 (0.19) | | | 0.375 | |  | | 0.19 (0.04) | | | 1.71E-06 | | 0.14 (5.45) | |
|  | Height | rs2928391; 10; 49667278; 10q11.22 | WDFY4 | intronic | A | SNP main | 0.27 | -16.2 (3.74) | 1.53E-05 |  | 0.26 | | -5.50 (2.80) | | | | 4.94E-02 | |  | 0.30 | | 16.4 (11.7) | | | 0.162 | |  | | -10.6 (2.61) | | | 4.89E-05 | | 0.00 (19.0) | |
|  |  |  |  |  |  | Interaction |  | 0.10 (0.02) | 1.30E-05 |  |  |  | 0.03 (0.02) | | | | 4.76E-02 | |  |  |  | -0.10 (0.07) | | | 0.191 | |  | | 0.07 (0.02) | | | 3.83E-05 | | 0.00 (18.5) | |
| CHR, chromosome; MA, minor allele; MAF, minor allele frequency; A test of heterogeneity (*P_het_*) was conducted; Q, Cochrane's Q value based on chi-squared statistics | | | | | | | |  |  |  |  | |  | | | |  | |  |  | |  | | |  | |  | |  | | |  | |  | |
| *The results of rs10760966 in replication stage 2 were calculated only 356 subjects in Ehime study and combined meta-analyses for rs10760966 were performed in 11,545 subjects | | | | | | | | |  |  |  | |  | | | |  | |  |  | |  | | |  | |  | |  | | |  | |  | |

| **Table S5** Results of association analyses for SBP considered interaction between BMI and known SNPs that were revealed by earlier experiences within the KARE project | | | | | | | | | | | | |  |  | |  |
| --- | --- | --- | --- | --- | --- | --- | --- | --- | --- | --- | --- | --- | --- | --- | --- | --- |
| **Trait** | **Interaction** | **SNP** | **CHR** | **Cytoband** | **Genes** | **MA** | **KARE (n=7,486)** | | | | | **Ref.** | | | |  |
|  |  |  |  |  |  |  | **MAF** | **^1^effect(se)_SNP_** | ***^2^P_SNP_*** | **^3^effect(se)_INT_** | ***^4^P_INT_*** |  |  |  |  |  |
| SBP | BMI | rs11916762 | 3 | 3q29 | OPA1 | A | 0.35 | 1.50 (2.16) | 0.486 | -0.08 (0.09) | 0.374 | [1] | | | |  |
|  |  | rs10804955 |  |  |  | T | 0.35 | 1.42 (2.16) | 0.511 | -0.07 (0.09) | 0.396 | [1] | | | |  |
|  |  | rs7628243 |  |  |  | G | 0.35 | 1.49 (2.16) | 0.489 | -0.08 (0.09) | 0.378 | [1] | | | |  |
|  |  | rs7620342 |  |  |  | G | 0.35 | 1.45 (2.16) | 0.501 | -0.08 (0.09) | 0.387 | [1] | | | |  |
|  |  | rs9868128 |  |  |  | T | 0.35 | 1.49 (2.16) | 0.489 | -0.08 (0.09) | 0.378 | [1] | | | |  |
|  |  | rs12696670 |  |  |  | G | 0.27 | 2.14 (2.29) | 0.349 | -0.10 (0.09) | 0.305 | [1] | | | |  |
|  |  | rs11925699 |  |  |  | A | 0.35 | 1.77 (2.16) | 0.412 | -0.09 (0.09) | 0.306 | [1] | | | |  |
|  |  | rs6797738 |  |  |  | A | 0.27 | 2.05 (2.29) | 0.372 | -0.09 (0.09) | 0.331 | [1] | | | |  |
|  |  | rs7646250 |  |  |  | T | 0.35 | 1.67 (2.16) | 0.438 | -0.09 (0.09) | 0.326 | [1] | | | |  |
|  |  | rs7646539 |  |  |  | G | 0.27 | 1.97 (2.29) | 0.390 | -0.09 (0.09) | 0.357 | [1] | | | |  |
|  |  | rs9851685 |  |  |  | C | 0.33 | 1.79 (2.22) | 0.419 | -0.10 (0.09) | 0.287 | [1] | | | |  |
|  |  | rs9837255 |  |  |  | T | 0.25 | 2.38 (2.39) | 0.320 | -0.11 (0.10) | 0.252 | [1] | | | |  |
|  |  | rs872663 |  |  |  | A | 0.28 | 2.47 (2.29) | 0.281 | -0.12 (0.09) | 0.192 | [1] | | | |  |
|  |  | rs9847250 |  |  |  | A | 0.26 | 1.68 (2.31) | 0.466 | -0.08 (0.09) | 0.403 | [1] | | | |  |
|  |  | rs6764269 |  |  |  | C | 0.35 | 1.03 (2.14) | 0.632 | -0.06 (0.09) | 0.489 | [1] | | | |  |
|  |  | rs6902041 | 6 | 6q26 | PARK2 | G | 0.37 | -2.07 (2.16) | 0.337 | 0.06 (0.09) | 0.511 | [2] | | | |  |
|  |  | rs995322 | 8 | 8p23.2 | CSMD1 | A | 0.36 | 0.96 (2.12) | 0.651 | 0.00 (0.09) | 0.975 | [3] | | | |  |
|  |  | rs11191548 | 10 | 10q24.32 | CYP17A1 | C | 0.25 | -1.33 (2.38) | 0.574 | 0.02 (0.10) | 0.823 | [4] | | | |  |
|  |  | rs381815 | 11 | 11p15.1 | PLEKHA7 | T | 0.19 | 2.13 (2.79) | 0.445 | -0.06 (0.11) | 0.574 | [4] | | | |  |
|  |  | rs17249754 | 12 | 12q21.33 | ATP2B1 | A | 0.37 | 0.89 (2.13) | 0.676 | -0.09 (0.09) | 0.288 | [3], [4] | | | |  |
|  |  | rs1378942 | 15 | 15q24.1 | CSK | A | 0.17 | -0.20 (2.81) | 0.944 | -0.05 (0.11) | 0.645 | [3], [4] | | | |  |
|  |  | rs12945290 | 17 | 17q24.2 | ARSG | C | 0.13 | -1.41 (3.12) | 0.651 | 0.01 (0.13) | 0.943 | [3] | | | |  |
| Ref., reference of previous studies for BP using KARE data; The number of references are [1] Jin et al. 2011 Am J Hypertens., [2] Jin et al. 2011 Clin Chim Acta., [3] Hong et al. 2010 J Hum Hypertens., [4] Hong et al. 2010 J Hum Genet. | | | | | | | | | | | | | | | |  |
| ^1^ Beta and s.e. for SNP main effect, ^2^ *P* value for SNP main effect, ^3^ Beta and s.e. for interactional effect, ^4^ *P* value for interactional effect; CHR, chromosome; MA, minor allele; MAF, minor allele frequency | | | | | | | | | | | | | | |  | |
